# Hidden in plain sight: how individual ADHD stakeholders have conflicting ideas about ADHD but do not address their own ambivalence

**DOI:** 10.1007/s00787-023-02290-w

**Published:** 2023-09-09

**Authors:** Myrte J. M. van Langen, Branko M. van Hulst, Sarah Durston

**Affiliations:** 1 University Medical Center Utrecht, Heidelberglaan 100, 3584 CX Utrecht, HP A01.126 (B01.106), Utrecht, The Netherlands; 2 LUMC Curium - Child and Adolescent Psychiatry, Leiden University Medical Centre, Leiden, The Netherlands

**Keywords:** ADHD, Attention-Deficit/Hyperactivity Disorder, Focus Groups, Ambivalence

## Abstract

Psychiatric classifications refer to clusters of behavioral symptoms. We know much about how psychiatric classifications are intended to be used in theory. Yet the scientific study of the *practice of classification* to date is limited. We aimed to explore how individuals navigate and make sense of the complexity surrounding an ADHD classification. We used thematic analysis to analyse stakeholder perspectives from seven focus groups: adults classified with ADHD, adolescents classified with ADHD, parents of children classified with ADHD, clinicians, researchers, teachers, and policy makers. We found seven themes in how stakeholders navigate the classification ADHD. Yet, what stood out was an overarching discursive pattern: individual stakeholders expressed highly ambivalent ideas about ADHD but did not address their own ambivalence. We suggest that promoting a social kinds perspective on ADHD may help us navigate the complexity and ambivalence associated with ADHD more competently.

**Supplementary Information:**

The online version contains supplementary material available at 10.1007/s00787-023-02290-w.

## Introduction

Psychiatric classifications, such as ADHD, are names that refer to clusters of behavioral symptoms [1, 2]. These names matter. A classification is often taken by a person to represent how their story is understood by a therapist. This in turn impacts the therapeutic alliance, a robust mediator of treatment outcome [3–7]. Moreover, the impact of classifications has come to stretch well beyond the realm of mental healthcare [8–10]. Psychiatric classifications indirectly shape the way we understand psychological differences in society.

We know much about how psychiatric classifications are intended to be used in theory. Yet the scientific study of the *practice of classification*, the way our use of classifications is realized in professional and daily life, is limited. We use the term ‘classification’ rather than ‘diagnosis’, as we focus specifically on the classifications assigned to individuals and not on the broader process of psychiatric diagnosis. From a theoretical perspective, we know that classifications were developed to increase reliability, validity and communication in diagnostic practice and research [11–14]. In addition, classification aims to provide an indication of prognosis and guide decisions regarding care and treatment allocated to affected individuals, including children with ADHD [11–15]. On the other hand, the increase in the prevalence of ADHD-classifications has raised questions on the reliability and validity. This increase has been argued to represent a social and cultural shift in the perception and acceptance of diversity among young people [16–19]. Moreover, the word ‘disorder’ can be taken to suggest that the term ADHD represents a stable and causal ‘core deficit’ in the functioning of the child, promoting fatalism and inappropriate interventions [18, 20–22].

Empirical studies of the effect of diagnostic classifications show that individuals with an ADHD classification report feeling more understood and recognized because of it. These studies find that classification can reduce the blameworthiness of difficulties [21, 23]. On the other hand, the negative stigma that is often associated with classifications such as ADHD [24] affects access to services, being treated justly and fairly, treatment adherence, resilience and advocacy, [25], all of which in turn affects the development of children in ways that are not yet understood. Concerns are also increasingly being raised about the failure of biomedical research to uncover definitive causes of and cures for DSM categories [12, 15, 16].

Despite the extensive theoretical and empirical literature, we know little about how individuals navigate and make sense of the complexity surrounding an ADHD classification. Our previous work shows different conflicts in how ADHD is explained by experts in psychoeducational materials [26], but does not provide information on how this is integrated and understood by ADHD stakeholders in practice. This integration of information can be linked to the concept of tinkering: the attempt to understand, integrate and negotiate the complexity of available knowledge and technologies to accomplish ‘good care’ [27].

In this study, we aimed to explore the *practice* of classification, by investigating how a broad set of stakeholders navigated the classification ADHD. The results may be useful in advising stakeholders how to better navigate a classificatory term such as ADHD ‘in the wild’. We analyzed perspectives on the ADHD classification from seven stakeholder groups: adults classified with ADHD, adolescents classified with ADHD, parents of children classified with ADHD, clinicians, researchers, teachers, and policy makers. We collected verbatim data from the seven focus group discussions on this topic and analysed the discussion prompted by our questions using thematic analysis. We hypothesized that perspectives on ADHD classification would vary both between stakeholder groups and between participants within a stakeholder group. However, we had no a-priori hypotheses on the nature of these perspectives and aimed to explore a broad range of different perspectives on ADHD classification.

## Method

### Procedure

In this qualitative study, we assessed how the classification ADHD is used and understood in daily practice. Over the course of 18 months, we organized seven focus groups with the following stakeholders: adults with ADHD, adolescents with ADHD, parents of children with ADHD, clinicians, researchers, teachers, and policy makers. There was a separate focus group for each of the stakeholder groups. We opted for homogeneous focus groups to gain insight into the different stakeholder perspectives and to stimulate conversation around similar experiences, without hierarchy or status differences. [28] We prompted discussion on the classification ADHD with a standardized set of questions (see *Data Collection* and Supplement 1). The Medical Ethical Committee of the University Medical Centre Utrecht judged that the overall research project did not require evaluation based on the Medical Research Involving Human Subject Act (WMO) and that it complied with the Netherlands Code of Conduct for Research Integrity. Reporting of the study methods and results was informed by the Consolidated Criteria for Reporting Qualitative Research (COREQ; 32).

### Participants

We identified seven stakeholder groups, each involved in, or subject to the process of diagnostic classification. Individuals who self-identified as a member of any of the seven groups were eligible to participate. Four to eight participants were recruited for each group, as recommended in the literature [28–30]. Several clinicians unexpectedly brought colleagues to this focus group, so we included a higher number of participants in this group (10). We used a variety of different recruitment methods: we invited stakeholders using online advertising and social media posts (purposive sampling), we invited individuals through our own network (convenience sampling) and asked interested participants to recruit within their own networks (snowball sampling). A general description of participants in each group is provided in Table 1.

**Table 1:** Participant demographics per focus group

	Participants	Number	Female/Male	Average Age	Age Min/Max
1	Adults with ADHD	5	4/1	39,8	23/52
2	Parents	4	4/0	50,0	44/60
3	Clinicians	10	7/3	46,5	28/65
4	Teachers	6	4/2	44,1	27/64
5	Researchers	6	4/2	31,7	26/34
6	Policy Makers	4	3/1	50,5	43/57
7	Adolescents with ADHD	4	2/2	14,5	12/17

### Data Collection

The first three focus groups (adults with ADHD, parents of children with ADHD and clinicians) were organized in conference rooms of the University Medical Centre Utrecht. Due to COVID-19 restrictions, we held the subsequent focus groups using the video-conference platform WebEx. As recommended in the literature [28], focus groups lasted approximately two hours. They included a 5–10 min introduction and a 15 min break. Audio recordings were made of each focus group meeting.

We designed a preliminary list of topics with questions for the focus groups. We discussed this list with colleagues from our broader research project on diagnostic labels (https://www.uu.nl/en/research/dynamics-of-youth/research/interdisciplinary-hubs/developmental-labels-the-good-the-bad-and-the-contested). Based on their feedback, we refined the topic list and designed the focus group manual provided in Supplement 1. This manual was used for all focus groups and served as a general guide for discussion.

Two researchers (BvH and MvL) moderated the focus groups. At the start of each session, we introduced the research project and urged participants to ask any lingering questions. All participants then signed for informed consent. We then started the recording and initiated the introduction round. Subsequently, we introduced the questions, as stated in the manual. During the focus groups, we encouraged stakeholders to talk freely and openly with one another. Participants were specifically instructed to discuss topics they found most relevant and ask each other questions to elucidate their answers. At the end of the session, a short debriefing took place, during which participants had the opportunity to ask questions and reflect on the focus group.

### Analysis

A detailed overview of our analysis plan is provided in Supplement 2. All focus groups were analyzed separately, before results were combined and integrated. We transcribed all focus group recordings verbatim. We imported the transcriptions into NVivo 12 Pro and carried out a thematic analysis. We identified the most important themes in each of the groups using a bottom–up approach without preconceived ideas or structures of what the data should represent. We used the coding method, as described by Corbin and Strauss [31], that includes three steps; open, axial and selective coding. In open coding, we broke up and described all textual data, in axial coding we created broader categories connecting open codes together and in selective coding we created large overarching themes that captured our core findings. MvL carried out the first two steps of the coding process, *open coding* and *axial coding* for each of the focus groups separately. For each separate focus group, she visualized the coding schemes in PowerPoint and wrote memos on the content of the data. The preliminary thematic structure of the data was also visualized in the PowerPoint and these presentations were used to guide discussion and exploration of the data during in-depth discussions between MvL and BvH. Memos were written solely as notes kept by MvL, to track ideas, thoughts or findings.

In preparation for the third step of *selective coding,* MvL relistened and reread each individual focus group and studied all coding schemes, memos, and notes. Subsequently, MvL and BvH integrated data and patterns across focus groups. To integrate analyses, we studied and compared which themes were present in all focus groups, and which themes were specific to one or a subset of focus groups. An overarching coding scheme was then constructed by combining the separate NVivo files, and the themes were further defined, named and described in Nvivo to support analyses and find relevant text excerpts.

## Results

We found a total of seven different themes in our focus group discussions. Four themes were present in most or all focus groups and three themes were specific to a (subset of) focus group(s). In addition, we found one discursive pattern that we will describe first, as it was present across the first four themes.

### Discursive pattern

#### Dormant ambivalence

We hypothesized that perspectives on ADHD classification would vary both between stakeholder groups and between participants in a particular stakeholder group. Unexpectedly, we identified a different pattern. We found that participants would endorse differing perspectives, even if these perspectives conflicted. So rather than choosing a ‘side’ in a particular debate, participants would agree with all ‘sides’ within a debate at different points during the conversation. Consequently, a single stakeholder would make conflicting statements in the course of a focus group. Interestingly, participants seemed to be largely unaware of this conflict. We call this phenomenon ‘dormant ambivalence’. Participants appeared to agree with opposing perspectives but did not actively address the opposition or ambivalence during the conversation. The data are lacking in statements such as ‘on one hand (….), but on the other hand (….). Instead, multiple realities appeared to exist simultaneously for participants, without participants discussing the extant contradictions.

### Themes present in all or most focus groups

#### ADHD says both nothing and a lot about a person

We started each of the focus groups with the following question: “What does having ADHD say about a person?”. A straightforward answer would have been to name the associated symptoms of hyperactivity, inattention and impulsivity. Yet, this was not the response we got. One of the first responses we received across all groups was a variation on the statement: “Having ADHD says nothing about a person”. Noticeably, this answer did not correspond with the data from the rest of the conversations. After participants noted that having ADHD meant nothing, they would often list a multitude of things having ADHD *does* say about a person. Occasionally, DSM criteria were mentioned, but other responses included: (1) having ADHD suggests that someone experiences difficulties or problems, (2) having ADHD suggests that a person deviates from the norm, (3) having ADHD suggests that a person needs additional help and support, (4) having ADHD means that someone has visited a clinician and received psychological assessment, and (5) having ADHD indicates that someone has altered brain structure or functioning. Exemplary quotes for this theme can be found in Table 2.

**Table 2:** ADHD says both nothing and a lot about a person

**Quote Focus group 1: Adults with ADHD** While introducing herself, one participant mentioned how important her ADHD diagnosis was to her, that a lot had fallen into place when she was classified and that it had given her a much better understanding of herself. When asked what having ADHD said about a person, she said ‘nothing’ and questioned what having ADHD said about her		
Participant 1.2	“A *lot fell into place for me and I thought, ah, now I understand a lot of things*”	
	(…)	
Moderator	“*What does having ADHD say about a person?*”	
	(…)	
Participant 1.2	“*Well, my first inkling is [to say] nothing. It actually says nothing. If I look at our society, and that is something I struggle with personally, we always need [to get] a classification first, [ascribe] a label or a box, before someone can get the right help. Because then I wonder, but what does it actually say about me?”*	
**Quote Focus group 3: Clinicians** One participant first stated that she could not say what ADHD says about a person, because it does not say anything. Yet subsequently, she noted that we diagnose ADHD when people get stuck and that the classification ADHD informs us on brain-functioning, citing the ADHD-brain		
Participant 3.0	“*So yes, what does it say about a person? I cannot answer that question at all, [it] says absolutely nothing.*”	
	(…)	
Participant 3.0	“*But we have agreed to diagnose ADHD if someone gets stuck in multiple areas [of life], but you can still have an ADHD-brain. At least that is what I would call it, having an ADHD brain.*”	
**Quote Focus group 2: Parents of children with ADHD** Similarly, one participant in this group said, within a single sentence, that ADHD means nothing and yet it also means that someone has certain characteristics		
Participant 2.2	*“Well technically [ADHD means] nothing, no. Certain characteristics, that a label has been attached to.”*	

#### The impact of the classification ADHD is both positive and negative

Throughout the focus groups, participants extensively discussed the advantages and disadvantages of having an ADHD classification. Positive aspects mentioned included: (1) it takes away blame from an individual child and stimulates the acceptance of diversity, (2) it provides clarity, (3) it explains why children behave the way they do and (4) it opens doors to support and treatment. In the same vein, participants discussed negative aspects of having an ADHD classification. Many of the disadvantages mentioned were direct contradictions to the advantages mentioned. These included: (1) having a classification might lead to stigma and stimulate focus on negative characteristics of a child. As such, it may lead to less acceptance of individual variation and lay blame with that individual. (2) The classification does not indicate what an individual needs and might be taken to suggest that all individuals with the classification require the same approach and treatment. (3) The diagnosis (participants usually referred to ADHD as a diagnosis rather than a classification) is vague, unclear, and unspecific. Every individual with a classification is different and knowing his or her classification does not help to understand an individual child. Exemplary quotes for this theme can be found in Table 3.

**Table 3:** The impact of the classification ADHD is both positive and negative

**Quote Focus group 1: Adults with ADHD** This participant resented the classification ADHD, because of the number of value-judgements she felt are attached to it. She felt the classification does not do her justice. Yet in a subsequent remark, she stated that if only she had known about having ADHD sooner, her life would have been a lot easier		
Participant 1.3	“*I think the term ADHD is horrible. I feel like it [the term ADHD] is totally wrong, because there are so many value judgements attached to it: [in a disparaging voice]’you are a little hyperactive today…’ All of that. While I think, can we please do something that does me justice! [disparaging voice:] ‘Oh, and I’m a bit ADHD too’*	
Participant 1.5	*[disparaging voice] “I am a little bit depressed too”*	
Participant 1.3	*“Yes everybody [goes] ‘I am a little bit hyperactive too*’, *I think it’s terrible, and then you get these discussions about getting rid of our stickers [labels], and I think, my goodness, if only I had known, my life would have been so much easier. So, that’s it really, I think [the term] ADHD is becoming increasingly empty.*”	
**Quote Focus group 2: Parents of children with ADHD** The quote below shows a similar contradiction, where one participant first said the classification ADHD leads to more help, money, and support from schools. Yet in a subsequent statement, the same participant remarked that the classification leads to people only looking for what is wrong with her child rather than for what he needs		
Participant 2.1	“*For me [the classification ADHD] only means that the school and other organizations are willing to help you with that particular bit [of the problem]. Without the label, they won’t. The label [only] means money, it does not change a thing about my child”*	
	(…)	
Participant 2.1	“*Yes, [they] only look at what is wrong with him, instead of what he needs”*	
**Quote Focus group 4: Teachers** One teacher mentioned that having an ADHD classification might lead to more understanding and acceptance of ADHD-related behavior. Then the same teacher stated that ADHD leads to a continued negative association with or negative evaluation of a child and their behavior		
Participant 4.5	*“For some people [it [ADHD] leads to] understanding, for some children it provides [more] understanding of their situation”*	
Moderator	“*[understanding] from themselves, from their parents, from their teachers?*”	
Participant 4.5	“*Well, from everyone I think”*	
Moderator	“*And how does this understanding work? Strange question perhaps, but how…*”	
Participant 4.5	“*Well, they know, or they can better place, where their behavior is coming from, or what causes it. That leads to a better understanding of the situation, so [understanding] of that some things don’t go well. And some do. And that you maybe feel you are extra special, so yeah, in that way you can gain understanding of your own behavior, as a child.”*	
	(…)	
Participant 4.5	“*But if you are that ‘ADHD-child’ who is always messing around in class, and if you are constantly referred to that way, then you can develop a very negative association [with ADHD] and a very negative self-image.”*	

#### Considering ADHD to be a category is both helpful and harmful

Across all groups, participants mentioned that the classification ADHD can function as a convenient shorthand to understand what an individual needs quickly. It indicates the need for a certain treatment or approach and helps parents, teachers, and clinicians to make an initial quick assessment of treatment options. Yet simultaneously, in all groups participants mentioned that the classification does not actually provide any information about an individual. They discussed that care should always be provided based on individual needs rather than based on a classification. In several groups, participants would criticize and even ridicule parents, clinicians and teachers who did, in fact, use the classification as a shorthand. In other words, participants stated that the classification can and should be used as a shorthand and at the same time criticized individuals around them who did so. Both perspectives are not necessarily mutually exclusive, but it was noticeable that participants did not attempt to actively integrate these perspectives. Exemplary quotes for this theme can be found in Table 4.

**Table 4:** Considering ADHD to be a category is both helpful and harmful

**Quote Focus group 1: Adults with ADHD** One participant argued that we should steer clear from labeling everyone, and that people should be allowed to simply be, without bringing in classifications or names. When the moderator tried to verify that we can tell people’s story without classifications, she described how she defines everyone in her family by their classification		
Participant 1.3	“*But [if] you want a name [label], you could also just say ‘I am human’*.”	
Participant 1.1	“*Yes, well, I would like that, but we are not at all ready for that as a society. There is already much, much more room for all the different colors and shapes [than there was]. But we are also taking that too far, in that everyone has to have a color or shape, while at a certain point we’ll get to… we’ll just let things be.*”	
Moderator	“*That [problems] can exist without a label?*”	
Participant 1.1	“*Without a label.*”	
Moderator	“*So, what your [Participant 1.3] question was, you say you are ‘human’, and someone asks’ what kind of human are you?’, and then one day, you will be able to tell your whole story, but you won’t need that label [ADHD] anymore? Is that possible?*”	
Participant 1.1	“*Yes, in our house, my son has autism, I have ADHD, there is nothing wrong with my daughter, but we say ‘you have has eczema, and dad is colorblind’. You know, so that we how we…*”	
**Quote Focus group 6: Policy Makers** In this quote, one of the policy makers described how an ADHD classification should serve as a road map to better determine how to handle and support someone. Yet, subsequently she stated that teachers should not use previous experiences or outdated stereotypes to handle or support children in their classrooms but should rather consider an individual child’s needs		
Participant 6.4	“*It [the classification] is a point of departure, and, actually, you should be given a map. The person who gives the diagnosis should give other people a roadmap. That way, we don’t just answer the question of whether it is ADHD or not, but it serves as a point of departure, of ‘okay we are doing this and this, and it means this for you, this for your teachers and this for your friends’. It can serve as a roadmap.”*	
	(…)	
Participant 6.4	““*Yes, and often I think that teachers have had experiences in the past, with another student who had a similar label, and that time specific things worked. So, then it is tempting and easy to think that it will be the same now, especially if it [the experience] was like five years ago, when we treated it [ADHD] in a more stereotypical way. Then you might have missed a few steps of what we are referring to; we are trying to stimulate development, and focus more on an individual [child].”*	

#### ADHD is rooted in the brain and in society, both as a cause and a consequence

We noted that discussions surrounding causality and consequences of ADHD were complex, confusing, and often difficult to follow. This was because ADHD was described both as a cause of problematic behaviors, and a consequence of these same behaviors. Simultaneously, ADHD was described as both a neurobiological and a societal problem. Participants noted that society leaves little room for children to develop freely. Children who deviate from the norm are quickly labeled. Society leaves little space for developmental variability and children are expected to excel and perform at a high level from a young age. Yet in most focus groups, participants also described ADHD as a disorder of the brain. The neurobiology of individuals with a classification was said to be different from the neurobiology of individuals without a classification. Participants referred to this phenomenon as an ‘ADHD brain’. It was difficult to pin down participants’ point of view in these discussions, as participants seemed to jump from one perspective to another, without acknowledging or interpreting the differences and similarities between perspectives. Exemplary quotes for this theme can be found in Table 5.

**Table 5:** ADHD is rooted in the brain and in society, both as a cause and as a consequence

**Quote Focus group 3: Clinicians** The conversation below gives an example of one such conversation. Participants agreed that children do not get enough time and space to fully develop. Yet they did not believe that this is a cause of ADHD. They then reiterated that children do not get the opportunity to mature and underline that society imposes certain expectations and norms on children, and that this may lead to children developing an impairment. Then cause and consequence were reversed and ADHD was discussed as the cause of impairment. Subsequently, in response to the question of whether ADHD is a cause or a description, one participant brought up the ADHD brain		
Participant 3.9	“*There is nobody here who disagrees with you that we should be attuning [our society] to the needs of those children.*”	
Participant 3.5	**“***No, but those children don’t get that time and space anymore.*”	
Participant 3.9	“*Well, that is the question, so you, you are more or less assuming that children develop ADHD from people not engaging with them properly.*”	
Participant 3.7	“*No, that is not true*”	
Participant 3.5	“*No, you don’t get ADHD from [how people engage with you]*”	
Participant 3.7	“*Children don’t get the time to grow up*”	
Participant 3.0	“*No, which improvements are needed with regard to the term ADHD? Nothing wrong with the term ADHD, I think, that’s roughly what we have said here. But we have to realize that in this society, in this moment, the demands we put on children, that…*”	
Participant 3.3	“*They lead to them dysfunctioning more quickly, to getting stuck….*”	
Participant 3.7	“*But that does not always need to be caused by ADHD*”	
Participant 3.6	“*No, but that is the tendency, [to ascribe it to ADHD]”*	
Moderator	“*Is ADHD a cause or a description?*”	
Participant 3.9	“*Yes, exactly*”	
Participant 3.0	“*Well, in my eyes, but we already talked about this at the beginning, I call that an ADHD-brain.*”	
**Quote Focus group 1: Adults with ADHD** This participant explained that individuals with ADHD often only struggle because their environment is not properly attuned to their needs. Yet he then went on to describe how ADHD is an engagement disorder that appears to be inherent to the individual and related to his/her ability to connect and disconnect their attention		
Participant 1.5	“*Because you struggle more with things… But people with ADHD don’t have to struggle more, it only works out that way because they are not in the right environment.”*	
	(…)	
Participant 1.5	“*I have a sort of personal hypothesis, that I can’t test, because I am no longer a researcher, [that] ADHD is much more of an engagement disorder. So, it literally is the connecting and disconnecting of attention, and I see [it] in many cases. If you look at hyperfocus, a bomb could literally explode behind you, but you stay focused, because you are engaged and your brain doesn’t disconnect anymore, it gets stuck. And sometimes it [isn’t] stuck and it will connect to anything because it doesn’t know, well, the reward-seeking part of the brain has something to do with it. I don’t know, I am no longer a researcher. But that is kind of how I explain it, it is an engagement disorder and it is just difficult to control what you attend to.*”	
**Quote Focus group 2: Parents of children with ADHD** This participant first explained that medication helps her son focus on his tests at school. Yet subsequently, she made a point of stating that it is ‘bizarre’ that we give children medication to change who they are and what they can and cannot do		
Participant 2.3	“*Yes, Ritalin, that is the solution. Oh ADD, well, then you’re given Ritalin, then everything is okay*”	
Participant 2.1	*“Well, but that’s not a..*”	
Participant 2.3	“*Maybe it does help him, I don’t know*”	
Participant 2.1	“*Yes, it does help my son, a lot, to only focus on his tests, while [he’s] taking the test. Instead of [thinking about] video games and those sorts of things*”	
	(…)	
Participant 2.1	“*But with children, we say, okay, so now we know that you are not a blue flower, you are a pink flower. So we give you pills, so that you can have blue flowers anyway. Well, that is bizarre, right? I think that is completely insane. [Why can’t we] just embrace that this child has pink flowers. It’s great right? It changes things up.”*	

## Themes specific to (a set of) stakeholder groups

### Adults and adolescents with ADHD

In the two stakeholder groups of adults and adolescents classified with ADHD, there was a specific focus on medication. This theme was extensively discussed in both groups and medication use was experienced differently by various participants. Participants were interested in each other’s experiences and clearly wanted to discuss the topic of medication. Noticeably, there was no in-depth discussion of the impact and implications of medication use in any of the other groups.

Participant 1.5: “*But, I solved that by saying, society benefits much more if I take Ritalin, and that is why I take it. Not because it makes me better, but because then I can just contribute more. And that is the reason I take it, because otherwise I would also be like, yes, it is actually unfair, but what is unfair about contributing more?*”.

Participant 7.2: “*I do have a question, for those of you who take pills, how do you guys feel about those pills?*”.

Participant 7.2: “*And sometimes I try to just pretend that I took my pills, because sometimes I find them a little bit annoying, because then I am suddenly very calm and serious.*”

### Parents and teachers

In the two stakeholder groups with parents and teachers, there was much focus on the quality of teaching and schooling. Parents extensively discussed their children’s and their own experiences with the school system and with teachers and noted many flaws in the system. Specifically, they discussed a lack of funding for appropriate support, inadequate teacher expertise regarding the specific needs of their child and a tendency to overlook individual children’s needs. Notably, the stakeholder group with teachers discussed similar topics. Teachers in this focus group were critical of the expertise of their fellow teachers and were highly critical of the lack of funding and flexibility in the school system to support children with special needs. Similar topics were occasionally mentioned in other focus groups, but to a much lesser extent.

Participant 2.1: “*I really, not once, but on multiple occasions, left the school crying because I couldn’t get through to them.*

Participant 2.2: “*Very frustrating, that powerlessness.*”

Participant 4.2: “*But it is all about money, it is all about getting that piece of paper (diploma) and how a student gets from A to B doesn’t really matter to them (the schools) at all.*”

### ADHD researchers

The stakeholder group with ADHD researchers was the only group where a clear meta-discussion of the utility and meaning of psychiatric classifications developed. The other groups mostly worked from the assumption that ADHD is a valid category and that we need to work out how to apply this category properly, whereas this was elaborately discussed (and disputed) in the group of ADHD researchers. Other groups elaborated on the direct implications of an ADHD classification, its advantages and disadvantages and when to use it. The researchers also discussed the utility of a classification for children and what these classifications mean, including concepts of reification and circular reasoning in psychiatry.

Participant 5.5: “*Yes, but I think indeed that it is a disadvantage that people sort of see it as, oh I have ADHD, so it is because of that… Then you start to see it as an explanation, which it isn’t really, of course, because it is actually more of a description of how a child behaves.*”

Participant 5.6: “*Well, I think that researchers themselves have slowly started to believe that it (ADHD) is a concrete thing… that is the thing about these terms, if they exist for a long time, they start to live a life of their own. And then that makes me think about my own neuroscientific research and many of you have also done this. On some level think that it also secretly plays a role in my thinking, that I make it more of a thing (ADHD) than it really is.”*

## Discussion

We carried out an exploratory thematic analysis of the perspectives of participants in seven focus groups of stakeholders on the classification ADHD. We aimed to explore the *practice of classification,* as opposed to the *theory of classification*. We found seven different themes in how stakeholders navigate the classification ADHD. Four themes were common to all or most stakeholder groups, while three themes were unique to a (subset of) focus group(s). The four themes common to all groups were: ADHD says both nothing and a lot about a person, the impact of the classification ADHD is both positive and negative, considering ADHD to be a category is both helpful and harmful and ADHD is rooted in the brain and in society, both as a cause and a consequence. Each of these links to various aspects of a broader discussion around the psychiatric classification ADHD, as outlined by Frances [12, 16], Werkhoven [32], Corrigan [8] and Stangl [25]. However, what stood out in our study was an overarching discursive pattern: participants expressed highly ambivalent ideas on ADHD, but made little or no reference to their ambivalence.

We hypothesized that perspectives on ADHD classification would vary both between stakeholder groups and between participants within a stakeholder group. However, we were left confused by the contradictory accounts from stakeholders, where they agreed with different sides of a debate sequentially. Conflicting accounts of ADHD were not debated between participants; rather, they were endorsed by the same individuals, with participants switching between perspectives as the discussion evolved. Ambivalence is a common phenomenon, defined as a state in which both positive and negative feelings are simultaneously associated with an object [33, 34]. However, the experience of conflict and ensuing negative affect determines whether objective ambivalence becomes subjective ambivalence (conflict is experienced) or remains dormant (conflict is not experienced) [34–36]. In our stakeholder groups, participants did not put the conflict between (their own) different perspectives into words. As such, we hypothesize that their *ambivalence* was *dormant,* in that participants were not aware of the conflicting aspects of their accounts.

This is relevant, as unacknowledged ambivalence may hinder the development of care practices for individuals with an ADHD classification. Mol, Moser and Pols note that “good care requires persistent tinkering in a world full of complex ambivalence and shifting tensions”. Managing ambivalence is, therefore, of paramount importance to ‘good care’ and requires adaptability and ‘attuned attentiveness’ [27, 37]. In our focus groups, we noted that participants do indeed tinker with their accounts of ADHD, as they attempted to combine and utilize different perspectives to navigate good care for ADHD. However, participants remained unaware of the conflicts that ensued. This aligns with findings from an earlier project, where we found similar conflicts in how ADHD is explained by experts in psychoeducational materials [26]. As such, we speculate that more competence in expressing and navigating ambivalence in our understanding of ADHD will result in better care practices.

In navigating the complexity of psychiatric classifications, a *social kinds* perspective may allow for more leeway than a *natural kinds* perspective. A natural kinds perspective suggests that classifications are representations of naturally existing categories which ‘cut nature at its seams’ [10, 38–40]. This approach leads to the (implicit and explicit) hypothesis that distinct biological mechanisms underly classifications, which are therefore fixed and lie within the individual. In contrast, a social kinds approach assumes that classifications are societal constructs that we have created and embraced [41, 42]. This allows for a more critical assessment of the current diagnostic system and suggests that we can decide if and when to classify experienced difficulties. Specifically, we surmised that participants in our focus groups (implicitly) operated largely from a natural kinds approach, where they believed classifications capture ‘true biological entities’ that cause problematic behaviours. Yet participants do attempt to integrate ideas from the social kinds approach into their rationale, and this leads to (undetected) conflict. Promoting a social kinds perspective, where the descriptive and a-theoretical nature of psychiatric classifications is stressed [43], may provide a framework for developing more awareness and competency in navigating the complexity of psychiatric classification.

We found three themes that did align with our hypothesis that perspectives on ADHD would vary between stakeholder groups. In the first of these, we found that youth and adults with ADHD often shared individual experiences with medication use. There was a lack of discussion about medication in the other focus groups, most noticeably in the focus groups of professional care providers. This may point to an underestimation of how, for individuals living with an ADHD classification, thoughts about ADHD classification and thoughts about medication are connected. In the second theme, we found that parents and teachers extensively discussed teaching and schooling, and their experiences with what they perceived to be a flawed system. This discussion highlights the importance of the school system in dealing with ADHD, and specifically of listening to those who are at the forefront of the diagnostic process. For both themes, the contributions of stakeholders with a lived experience underline important themes that may otherwise be missed. The third focus group-specific theme was found among ADHD researchers and revolved around a conceptual discussion of the ADHD classification [17, 44, 45]. This theme addressed the ongoing scientific discussion on the validity and utility of diagnostic classifications [12, 16, 17, 44, 45]. This discussion has been ongoing among Dutch researchers for numerous years, yet our results suggest that this debate has not yet spread beyond the academic environment.

Overall, we found conflicts in the way stakeholders understand ADHD that stakeholders themselves seemed unaware of. If we can encourage more awareness and competence in expressing and navigating the ambivalence associated with an ADHD classification, this may ultimately lead to better care practices.

## Limitations

A first limitation in our study is that participants were not representative of all stakeholders in ADHD. Although we attempted to invite stakeholders with different backgrounds and perspectives, selection bias was introduced by (of necessity) including only individuals willing to participate. This selection bias is evident in both age and ethnicity and may have been exacerbated by the recruitment of participants through our own network. Moreover, our sample had an overrepresentation of women, therefore concealing any gender differences in perspectives on ADHD. However, we were able to probe a variety of different perspectives and in this sense our sample was informative for this exploratory analysis. A second limitation is that we did not discuss our results with participants. This could be highly relevant to a follow-up study, as it may well be interesting and informative to ask participants to reflect on their dormant ambivalence.

## Implications and future directions

The conflicts we found in stakeholders’ understanding of ADHD highlights the need to encourage more awareness and competence in expressing and navigating the ambivalence associated with the classification. The conflicts were related to subtle, but relevant misunderstandings in how we discuss and communicate about ADHD. We need to develop clearer communication about what we do and do not know about psychiatric classifications and what they do and do not mean. Promoting a social kinds perspective, where the descriptive and a-theoretical nature of psychiatric classifications is stressed [43], may provide a framework for doing so. Moreover, knowledge of the nature of our psychiatric classifications should extend beyond the academic community. It should be shared, discussed and, most importantly, interpreted at the societal level, with all stakeholders involved.

Future research could, therefore, address whether stakeholders are aware of their own ambivalence surrounding the ADHD classification and if so, how they interpret it. By asking them directly about the conflicts in their accounts, we may gain a better understanding of how they have come to understand ADHD. For example, we could ask them how they understand ADHD as both a definition and a cause of behaviors; we could ask how the advantages and disadvantages directly opposing one another compare; and we could ask how a classification can guide our understanding of an individual, while simultaneously not saying much about the individual. Subsequent focus groups or individual interviews could provide answers to such questions.

### Supplementary Information

Below is the link to the electronic supplementary material.

Supplementary file1 (DOCX 16 KB)

Supplementary file2 (DOCX 18 KB)

Supplementary file3 (DOCX 60 KB)

Supplementary file4 (DOCX 54 KB)
