# Supplementary material for: Hidden in plain sight: how individual ADHD stakeholders have conflicting ideas about ADHD but do not address their own ambivalence
Source: Eur Child Adolesc Psychiatry. 2023 Sep 9;33(6):1921–33. doi: 10.1007/s00787-023-02290-w (PMC11211115; doi:10.1007/s00787-023-02290-w)
Supplement: Supplementary file 1 — Supplementary file1 (DOCX 16 KB) [file 787_2023_2290_MOESM1_ESM.docx]

**Supplement 1: Script for focus groups**

1. Introduction focus group (+/- 10 minutes)
   - Short introduction of the research project. (*We want to stress that our aim is to map different perspectives on ADHD. The goal is therefore not to the reach consensus when answering the questions. Differing opinions, experiences and stories are interesting to us).*
   - Has everyone had the opportunity to read the information letter and the consent form?
   - Are there any questions about the information letters or the consent form?
   - Moment to sign the consent forms.
2. Discussion – Part 1 (+/- 45 minutes).

(*Our goal is to ensure that participants can speak openly and freely about their ideas of, perspectives on, and experiences with ADHD. The participants should be guiding the conversation and introducing the topics that are to be discussed. If necessary, the moderators can ask additional questions or redirect the conversation. Below we present the questions that we introduce during the focus groups).*

- **Topic 1: How do we understand ADHD?**
  - What does having ADHD say about a person?
  - How do you prefer to talk about ADHD? Do you prefer to call it a diagnosis, classification, label or term?
- **Topic 2: Effect of the diagnosis ADHD**
  - What effect does the diagnosis ADHD have on the life of a child/adolescent/adult?
  - (if only advantages are mentioned): What are the disadvantages of receiving the label ADHD?
  - (if only disadvantages are mentioned): What are the advantages of receiving the label ADHD?

1. Break (+/- 15 minutes)
2. Discussion – Part 2 (+/- 45 minutes)

- **Topic 3: Effect of ‘the term’ ADHD (in a societal context).**
  - When do you talk about or use (the term) ADHD? (In what context?)
  - In your context, what are the advantages of using the term ADHD?
  - In your context, what are the disadvantages of using the term ADHD?
- **Topic 4: alternatives/improvement in the system?**
  - In current practice, we give children a diagnosis that has disadvantages, as well as advantages. Are there any improvements that can be made to the use of the term ADHD?
- Have we skipped over or forgotten any important questions or topics?

1. Ending (+/- 10 minutes)
   - Thank you for your active participation in this focus group!
   - How did you experience the discussion?
   - Are there any questions about the continuation of the study?
