# Supplementary material for: Hidden in plain sight: how individual ADHD stakeholders have conflicting ideas about ADHD but do not address their own ambivalence
Source: Eur Child Adolesc Psychiatry. 2023 Sep 9;33(6):1921–33. doi: 10.1007/s00787-023-02290-w (PMC11211115; doi:10.1007/s00787-023-02290-w)
Supplement: Supplementary file 2 — Supplementary file2 (DOCX 18 KB) [file 787_2023_2290_MOESM2_ESM.docx]

**Supplement 2: Focus group Analyses**

Below we describe the steps taken during the analyses and interpretation of the focus group data. For the first part of the analyses (step 1-4), we studied each focus group individually.

In the second part, we integrated the results into an overarching thematic structure. Within this thematic structure, we discussed all themes in the data and how these themes related to each other. We also discussed any themes that were specific to one or several focus groups.

***Part 1: Analyses of individual focus groups***

1. MvL transcribed all focus group recordings verbatim and imported the transcriptions into NVivo 12 Pro.
2. MvL analysed each of the transcriptions using a bottom-up approach, without preconceived ideas or structures of how the data would be represented. Each focus group was analysed separately, in a new NVivo file. The analyses consisted of a number of steps.
   1. The first half of each focus group was analysed using open coding; each relevant text excerpt was given a code that summarized its contents. Text excerpts about the same topics were grouped under the same code.
   2. After coding the first half of the group, the file contained roughly 100-150 codes. These codes were then grouped into axial codes; open codes with similar content were grouped together. This provided a more elaborate coding hierarchy, with codes split up into overarching topic groups.
   3. After the first half was sorted into axial codes, the second half of the focus group was also analysed using open codes. Text excerpts fitting previously discussed topics were placed within the pre-existing coding hierarchy. Text excepts addressing new topics were given new open codes.
   4. After completing open coding of the second half of the focus group, the axial coding hierarchy was reassessed, adding new codes into the hierarchy and creating additional axial codes when necessary.
   5. The final coding tree was then reassessed and checked for correctness.
3. MvL then visualized the coding hierarchy in a powerpoint presentation and wrote memos about the aspects of the focus groups and axial coding that stood out most.
4. During in-depth discussions between BvH and MvL, the axial coding was explored and discussed. These meetings were also used to discuss preliminary ideas about the interpretation of the analyses. MvL reassessed and added to the written memos after each of the discussion meetings.

***Part 2: Integration of the analyses***

1. MvL listened to and read along with the transcription of each of the focus groups over the course of four days
   1. Day 1: focus groups 1 and 2
   2. Day 2: focus groups 3 and 4
   3. Day 3: focus group 5
   4. Day 4: focus groups 6 and 7

This permitted MvL to refamiliarize herself with all the information discussed in the groups. MvL kept memos of information and ideas that stood out.

1. MvL reread the coding hierarchy, powerpoint presentations and memos for each of the focus groups. Based on all collected information, she integrated the overarching themes of each of the groups. She then combined the coding schemes in Nvivo to support analyses and find relevant text excerpts. Each theme was given a title and elaborately described in a document. After developing this overarching thematic structure, MvL also described those themes that were relevant to only one or several focus groups. These themes were described in a similar fashion.
2. The preliminary thematic structure was read and assessed by BvH. During subsequent in-depth discussions, MvL and BvH, integrated the thematic structure further and refined descriptions. During back-and-forth commentary and weekly discussion meetings, the thematic structure was written up into the Results section.
