# Supplementary material for: Hidden in plain sight: how individual ADHD stakeholders have conflicting ideas about ADHD but do not address their own ambivalence
Source: Eur Child Adolesc Psychiatry. 2023 Sep 9;33(6):1921–33. doi: 10.1007/s00787-023-02290-w (PMC11211115; doi:10.1007/s00787-023-02290-w)
Supplement: Supplementary file 3 — Supplementary file3 (DOCX 60 KB) [file 787_2023_2290_MOESM3_ESM.docx]

**Supplement 3: Overview Dutch Quotes and English Translations**

| ***Theme 1: ADHD says both nothing and a lot about a person*** | | |
| --- | --- | --- |
| *Quote Focus group 1: Adults with ADHD*  While introducing herself, one participant mentioned how important her ADHD diagnosis was to her, that a lot had fallen into place when she was classified and that it had given her a much better understanding of herself. When asked what having ADHD said about a person, she said ‘nothing’ and questioned what having ADHD said about her. | | |
|  |  |  |
| Participant 1.2 | “A *lot fell into place for me and I thought, ah, now I understand a lot of things*” | |
|  | (…) |  |
| Moderator | “*What does having ADHD say about a person?*” | |
|  | (…) |  |
| Participant 1.2 | “*Well, my first inkling is [to say] nothing. It actually says nothing. If I look at our society, and that is something I struggle with personally, we always need [to get] a classification first, [ascribe] a label or a box, before someone can get the right help. Because then I wonder, but what does it actually say about me?”* | |
|  |  |  |
|  | Participant 1.2 | “E*r viel voor mij veel op z’n plek, dat ik dacht, ah en nu begrijp ik heel veel dingen”* |
|  |  | (…) |
|  | Moderator | “*Wat zegt het hebben van ADHD over een persoon?*” |
|  |  | (…) |
|  | Participant 1.2 | “*Ja, mijn eerste gevoel is uh niks, eigenlijk zegt het niks ….. uhm, als ik , als ik kijk naar onze maatschappij en dat is wat ik persoonlijk zelf lastig vind, is dat er altijd eerst een classificatie, dus een labeltje of een hokje nodig is voordat iemand de juiste hulp krijg …. Want dan denk ik ja, wat zegt het nou over mij?*” |
|  |  |  |
| *Quote Focus group 3: Clinicians*  One participant first stated that she could not say what ADHD says about a person, because it does not say anything. Yet subsequently, she noted that we diagnose ADHD when people get stuck and that the classification ADHD informs us on brain-functioning, citing the ADHD-brain. | | |
|  |  | |
| Participant 3.0 | “*So, what does it say about a person? I cannot answer that question at all, [it] says absolutely nothing.*” | |
|  | (…) |  |
| Participant 3.0 | “*But we have agreed to diagnose ADHD if someone gets stuck in multiple areas [of life], but you can still have an ADHD-brain. At least that is what I would call it, having an ADHD brain.*” | |
|  |  |  |
|  | Participant 3.0 | “*Dus ja, wat zegt het over een person? Ik kan … Ik kan helemaal die vraag helemaal niet beantwoorden, zegt helemaal niets*” |
|  |  | (…) |
|  | Participant 3.0 | “*Maar we hebben wel afgesproken dat je ADHD vaststelt als iemand vastloopt he, op, op verschillende gebieden, maar je kan nog steeds een een ADHD-brein hebben, ten minste ik zou dat dan wel zo noemen, een ADHD-brein hebben..*” |
|  |  |  |
| *Quote Focus group 2: Parents of children with ADHD*  Similarly, one participant in this group said, within a single sentence, that ADHD means nothing and yet it also means that someone has certain characteristics. | | |
|  |  |  |
| Participant 2.2 | *“Well technically [ADHD means] nothing, no. Certain characteristics, that a label has been attached to.”* | |
|  |  |  |
|  | Participant 2.2 | *“Nou in principe niets, nee. Bepaalde eigenschappen, waar een labeltje aan gehangen is.”* |

| ***Theme 2: The impact of the classification ADHD is both positive and negative*** | | |
| --- | --- | --- |
| *Quote Focus group 1: Adults with ADHD*  This participant resented the classification ADHD, because of the number of value-judgements she felt are attached to it. She felt the classification does not do her justice. Yet in a subsequent remark, she stated that if only she had known about having ADHD sooner, her life would have been a lot easier. | | |
|  |  |  |
| Participant 1.3 | “*I think the term ADHD is horrible. I feel like it [the term ADHD] is totally wrong, because there are so many value judgements attached to it: [in a disparaging voice] ’you are a little hyperactive today…’ All of that. While I think, can we please do something that does me justice! [disparaging voice:] ‘Oh, and I’m a bit ADHD too’* | |
| Participant 1.5 | [disparaging voice] “*I am a little bit depressed too*” | |
| Participant 1.3 | *“Yes everybody [goes] ‘I am a little bit hyperactive too*’, *I think it’s terrible, and then you get these discussions about getting rid of our stickers [labels], and I think, my goodness, if only I had known, my life would have been so much easier. So, that’s it really, I think [the term] ADHD is becoming increasingly empty.*” | |
|  |  |  |
|  | Participant 1.3 | “I*k vind de term ADHD afschuwelijk. Ik vind hem echt helemaal verkeerd (ja) omdat er zoveel waardeoordelen aan hangen (stemmetje), jij bent ook weer een beetje dr.. Allemaal dat (gelach), terwijl ik denk, laten we in godsnaam iets doen wat recht doet aan mij. Ja, even in dit geval he uh, uh en ADHD dat heeft ook het jongetje in de st.. en oh, ik heb ook een beetje ADHD (gelach)*” |
|  | Participant 1.5 | “*Ik ben ook een beetje depressief.”* |
|  | Participant 1.3 | “*Iedereen hee.. ik ben ook een beetje druk, ah, ik vind het zo erg en dan zie je die discussies, laten we die stickers eens weghouden, en dan denk ik, my goodness, had ik het geweten, he, dan had ik een veel makkelijker leven gehad, dus, dat is eigenlijk, ik vind ADHD een heel erge leegte worden, zo onderhand.”* |
|  |  |  |
| *Quote Focus group 2: Parents of children with ADHD*  The quote below shows a similar contradiction, where one participant first said the classification ADHD leads to more help, money, and support from schools. Yet in a subsequent statement, the same participant remarked that the classification leads to people only looking for what is wrong with her child rather than for what he needs. | | |
|  |  | |
| Participant 2.1 | “*For me [the classification ADHD] only means that the school and other organizations are willing to help you with that particular bit [of the problem]. Without the label, they won’t. The label [only] means money, it does not change a thing about my child”.* | |
|  | (…) |  |
| Participant 2.1 | “*Yes, [they] only look at what is wrong with him, instead of what he needs*” | |
|  |  |  |
|  | Participant 2.1 | “*Voor mij zegt het alleen maar dat uh de school uh de school en de instanties bereid zijn jou te helpen op dat stukje, zonder dat labeltje, doen ze dat namelijk niet. (D24: geld, herhalen allemaal ‘geld’). Dat label zegt geld, het verandert niks aan mijn kind.”* |
|  |  | (…) |
|  | Participant 2.1 | “*ja, der wordt veel meer gekeken van wat heeft ie in plaats van wat hebben ze nodig.”* |
|  |  |  |
| *Quote Focus group 4: Teachers*  One teacher mentioned that having an ADHD classification might lead to more understanding and acceptance of ADHD-related behavior. Then the same teacher stated that ADHD leads to a continued negative association with or negative evaluation of a child and their behavior. | | |
|  |  |  |
| Participant 4.5 | *“For some people [it [ADHD] leads to] understanding, for some children it provides [more] understanding of their situation”.* | |
| Moderator | “*[understanding] from themselves, from their parents, from their teachers?*” | |
| Participant 4.5 | “*Well, from everyone I think”* | |
| Moderator | “*And how does this understanding work? Strange question perhaps, but how…*” | |
| Participant 4.5 | “*Well, they know, or they can better place, where their behavior is coming from, or what causes it. That leads to a better understanding of the situation, so [understanding] of that some things don’t go well. And some do. And that you maybe feel you are extra special, so yeah, in that way you can gain understanding of your own behavior, as a child*.” | |
|  | (…) |  |
| Participant 4.5 | “*But if you are that ‘ADHD-child’ who is always messing around in class, and if you are constantly referred to that way, then you can develop a very negative association [with ADHD] and a very negative self-image.”* | |
|  |  |  |
|  | Participant 4.5 | “*Voor sommige mensen begrip, voor sommige kinderen geeft het begrip in h.. hun eigen situatie”* |
|  | Moderator | “*Van zichzelf van ouders van leerkrachten?”* |
|  | Participant 4.5 | “*Ja, eigenlijk van iedereen denk ik.”* |
|  | Moderator | *“En en hoe werkt dat begrip? Rare vraag, maar hoe..”* |
|  | Participant 4.5 | “*Nou, dan ze uhm, weten wa.. of kunnen plaatsen waar hun gedrag door veroorzaakt wordt, of waar het vandaan komt, maar uh waardoor je dus meer begrip krijg voor een situatie, dus voor dingen die niet goed gaan, of die wel uh, goed gaan, dat je het misschien, ja extra bijzonder vindt, of ja op die manier eigenlijk en dus ook inzicht in je eigen gedrag, als kind.”* |
|  |  | (…) |
|  | Participant 4.5 | “*Maar als je altijd die ADHDer bent die loopt te rotzooien in de klas en als dat ook continue zo benoemd wordt uhm, dan kan je daar heel heel negatief uh associatie en een heel negatief zelfbeeld van krijgen.”* |
|  |  |  |

| ***Theme 3: Considering ADHD to be a category is both helpful and harmful*** | | |
| --- | --- | --- |
| *Quote Focus group 1: Adults with ADHD*  One participant argued that we should steer clear from labeling everyone, and that people should be allowed to simply be, without bringing in classifications or names. When the moderator tried to verify that we can tell people’s story without classifications, she described how she defines everyone in her family by their classification. | | |
|  |  |  |
| Participant 1.3 | “*But [if] you* *want a name [label], you could also just say ‘I am human’*.” | |
| Participant 1.1 | “*Yes, well, I would like that, but we are not at all ready for that as a society. There is already much, much more room for all the different colors and shapes [than there was]. But we are also taking that too far, in that everyone has to have a color or shape, while at a certain point we’ll get to.. we’ll just let things be.*” | |
| Moderator | “*That [problems] can exist without a label?*” | |
| Participant 1.1 | “*Without a label.*” | |
| Moderator | “*So, what your [Participant 1.3] question was, you say you are ‘human’, and someone asks ’what kind of human are you?’, and then one day, you will be able to tell your whole story, but you won’t need that label [ADHD] anymore? Is that possible?*” | |
| Participant 1.1 | “*Yes, in our house, my son has autism, I have ADHD, there is nothing wrong with my daughter, but we say ‘you have has eczema, and dad is colorblind’. You know, so that’s how we..*” | |
|  |  |  |
|  | Participant 1.3 | “*maar je wilt wel een naam, dan kun je ook zeggen ik ben mens’* |
|  | Participant 1.1 | “*Ja, maar das. Nou ja dat wil ik, dat wil ik wel... (praten door elkaar) maar daar zijn we als samenleving nog lang niet aan toe. En der is al heel erg veel, veel meer ruimte voor voor alle kleurtjes (ja) en vormpjes, uhm, maar daar slaan we ook weer in door, dat iedereen een kleurtje of een vormpje moet hebben, maar op een gegeven moment komt er wel denk ik wel, so.. soort van dat het er mag zijn.”* |
|  | Moderator | “*Dat ’t er mag zijn zonder naam.”* |
|  | Participant 1.1 | “*Zonder naam..”* |
|  | Moderator | *“Dus, wa wa wat jouw vraag was, mens en dan vraagt iemand door welke, nou wat voor mens ben je, en uiteindelijk (ja) kan je je hele verhaal vertellen en dan heb je niet meer die naam nodig. Dat dat ka..”* |
|  | Participant 1.1 | “*Ja, bij bij bij ons thuis, kijk mijn mijn zoon heeft autisme, ik heb ADHD, dus wij, wij, mij mijn dochter heeft niks, maar die noemt.. die heeft dan, jij hebt eczeem en papa is kleurenblind. Weet je, du dus zo geven wij (gelach)”* |
|  |  |  |
| Quote Focus group 6: Policy Makers  In this quote, one of the policy makers described how an ADHD classification should serve as a road map to better determine how to handle and support someone. Yet, subsequently she stated that teachers should not use previous experiences or outdated stereotypes to handle or support children in their classrooms but should rather consider an individual child’s needs. | | |
|  |  | |
| Participant 6.4 | “*It [the classification] is a point of departure, and, actually, you should be given a map. The person who gives the diagnosis should give other people a roadmap. That way, we don’t just answer the question of whether it is ADHD or not, but it serves as a point of departure, of ‘okay we are doing this and this, and it means this for you, this for your teachers and this for your friends’. It can serve as a roadmap.*” | |
|  | (…) |  |
| Participant 6.4 | ““*Yes, and often I think that teachers have had experiences in the past, with another student who had a similar label, and that time specific things worked. So, then it is tempting and easy to think that it will be the same now, especially if it [the experience] was like five years ago, when we treated it [ADHD] in a more stereotypical way. Then you might have missed a few steps of what we are referring to; we are trying to stimulate development, and focus more on an individual [child].”* | |
|  |  |  |
|  | Participant 6.4 | “*Ik ook, het is een soort vertrekpunt, en eigenlijk zal je, weleens de kaart mee moeten krijgen, zodat andere mensen ook okay, de he, degene die dan de diagnose heeft gesteld dat er een soort van meteen een routekaart of iets is, waardoor uh, niet alleen maar okay, i... ik, krijg de vraag is het ADHD of niet, effe heel uh ongechargeerd, nou, dat is het, je hebt vr.. antwoord op je vraag uh, uh alsjeblieft, maar dat daar dan als een vertrekpunt is van okay, nou dan gaan we hier en hier en dan betekent het d (…) Ja, voor jou, voor je leerkrachten voor je vrienden, uh, der is een soort roadmap..”* |
|  | (…) |  |
|  | Participant 6.4 | “*Ja, en volgens mij is het ook wel vaak dat leraren vaak dan zo van in het verleden ervaringen hebben gehad uhm, met een andere leerling die ook een vergelijkbaar labeltje had en toen werkte dit of dat, dus, dan is het natuurlijk ook verleidelijk of makkelijk om te denken van dat zal nu ook wel zo zijn, en zeker als een kind de tijd, he als het vijf jaar geleden is ofzo en het toen wat meer stereotiep mee om werd gegaan, dan ja, dan heb je misschien een aantal van die stappen gemist waar jullie het over hebben, van wij proberen ontwikkeling uh te krijgen, dat je meer naar het individu kijkt”* |
|  |  |  |

| ***Theme 4 ADHD is rooted in the brain and in society, both as a cause and as a consequence*** | | |
| --- | --- | --- |
| Quote Focus group 3: Clinicians  The conversation below gives an example of one such conversation. Participants agreed that children do not get enough time and space to fully develop. Yet they did not believe that this is a cause of ADHD. They then reiterated that children do not get the opportunity to mature and underline that society imposes certain expectations and norms on children, and that this may lead to children developing an impairment. Then cause and consequence were reversed and ADHD was discussed as the cause of impairment. Subsequently, in response to the question of whether ADHD is a cause or a description, one participant brought up the ADHD brain. | | |
|  |  |  |
| Participant 3.9 | “*There is nobody here who disagrees with you that we should be attuning [our society] to the needs of those children.*” | |
| Participant 3.5 | **“***No, but those children don’t get that time and space anymore.*” | |
| Participant 3.9 | “*Well, that is the question, so you, you are more or less assuming that children develop ADHD from people not engaging with them properly.*” | |
| Participant 3.7 | “*No, that is not true*” | |
| Participant 3.5 | “*No, you don’t get ADHD from [how people engage with you]*” | |
| Participant 3.7 | “*Children don’t get the time to grow up*” | |
| Participant 3.0 | “*No, which improvements are needed with regard to the term ADHD? Nothing wrong with the term ADHD, I think, that’s roughly what we have said here. But we have to realize that in this society, in this moment, the demands we put on children, that...*” | |
| Participant 3.3 | “*They lead to them dysfunctioning more quickly, to getting stuck...*” | |
| Participant 3.7 | “*But that does not always need to be caused by ADHD*” | |
| Participant 3.6 | “*No, but that is the tendency, [to ascribe it to ADHD]”* | |
| Moderator | “*Is ADHD a cause or a description?*” | |
| Participant 3.9 | “*Yes, exactly*” | |
| Participant 3.0 | “*Well, in my eyes, but we already talked about this at the beginning, I call that an ADHD-brain.*” | |
|  |  |  |
|  | Participant 3.9 | “*Der is het hier niemand met je oneens dat we ons moeten afstemmen op de behoefte van die kinderen.*” |
|  | Participant 3.5 | “*Nee, maar daar die die die die die die uh die kinderen krijgen daar de tijd en de ruimte niet meer voor.*” |
|  | Participant 3.9 | “*Nou ja, dat is, d.. dd.. dat is maar de vraag, dus ja, je je veronderstelt min of meer dat zeg maar uhuh, dat je ADHD krijgt van niet goed benaderd worden*.” |
|  | Participant 3.7 | “*Nee, dat is echt niet waar.*” |
|  | Participant 3.5 | “*Nee, je krijgt het er niet van.*” |
|  | Participant 3.7 | “*Je krijgt de tijd niet, om te rijpen.*” |
|  | Participant 3.0 | “*Nee, welke verbeteringen in ten behoeve van de term ADHD, niks mis met de term ADHD, volgens mij, was dat volgens mij wat we ongeveer hier gezegd hebben, (die is er), alleen uhm, uh we moeten ons beseffen dat in deze maatschappij, hoe die op dit moment uh uh is, uh, de eisen zo gesteld worden aan uh kinderen, dat uhm, uh.*” |
|  | Participant 3.3 | “*Dat je eerder uitvalt ,(ja) vastloopt.*.” |
|  | Participant 3.7 | “*Maar niet dat hoeft niet altijd als oorzaak ADHD te hebben.*” |
|  | Participant 3.6 | “*Nee, maar de de neiging.*.” |
|  | Moderator | “*Is, is ADHD een oorzaak of een beschrijving?”* |
|  | Participant 3.9 | “*Ja, precies.*” |
|  | Participant 3.0 | “*Nou ja, we.. in mijn ogen, maar dat dat daar hadden we het in het begin al over, hadden we het over, hadden we het over, ik noem dat dan toch een een een een ADHD-brein..*” |
|  |  |  |
| Quote Focus group 1: Adults with ADHD  This participant explained that individuals with ADHD often only struggle because their environment is not properly attuned to their needs. Yet he then went on to describe how ADHD is an engagement disorder that appears to be inherent to the individual and related to his/her ability to connect and disconnect their attention. | | |
|  |  | |
| Participant 1.5 | *“Because you struggle more with things… But people with ADHD don’t have to struggle more, it only works out that way because they are not in the right environment.”* | |
|  | *(…)* |  |
| Participant 1.5 | “*I have a sort of personal hypothesis, that I can’t test, because I am no longer a researcher, [that] ADHD is much more of an engagement disorder. So it literally is the connecting and disconnecting of attention, and I see [it] in many cases. If you look at hyperfocus, a bomb could literally explode behind you, but you stay focused, because you are engaged and your brain doesn’t disconnect anymore, it gets stuck. And sometimes it [isn’t] stuck and it will connect to anything because it doesn’t know, well, the reward-seeking part of the brain has something to do with it. I don’t know, I am no longer a researcher. But that is kind of how I explain it, it is an engagement disorder and it is just difficult to control what you attend to.”* | |
|  | Participant 1.5 | “*Want je hebt meer moeite met dingen, maar ADHDers die hoeven niet meer moeite met dingen te hebben, alleen vaak resulteert het wel op die manier, omdat ze dan niet in de juiste omgeving zitten of niet..*” |
|  | (…) |  |
|  | Participant 1.5 | “*ik heb nu en soort van uh, persoonlijke hypothese, ja die ik niet kan testen, want ik ben geen wetenschapper meer, uhm, dat ADHD veel meer een engagement disorder is, dus het is letterlijk het het koppelen en loskoppelen van aandacht, en ik zie het met heel veel dingen, a.. als je kijkt naar de hyperfocus, der kan een bom achter je ontploffen en je bent nog steeds geconcentreerd, want je bent gewoon bezig, jouw’n brein die koppelt niet meer los, als het eenmaal vast zit en soms dan zit het vast en dan koppelt het overal naartoe los, omdat het gewoon niet weet wanneer het nou ja, net als misschien ook met dat reward seeking gedeelte in je hersenen heeft, misschien ergens mee te maken. Ik weet het niet, ik ben geen wetenschapper meer. Maar, dat is hoe ik het nu een beetje uitleg van, het is een engagement stoornis, het is gewoon moeilijk om te reguleren waar je aan vasthoudt,..*” |
|  |  |  |
| Quote Focus group 2: Parents of children with ADHD.  This participant first explained that medication helps her son focus on his tests at school. Yet subsequently, she made a point of stating that it is ‘bizarre’ that we give children medication to change who they are and what they can and cannot do. | | |
|  |  |  |
| Participant 2.3 | “*Yes, Ritalin, that is the solution. Oh ADD, well, then you’re given Ritalin, then everything is okay*” | |
| Participant 2.1 | *“Well, but that’s not a...*” | |
| Participant 2.3 | “*Maybe it does help him, I don’t know*” | |
| Participant 2.1 | “*Yes, it does help my son, a lot, to only focus on his tests, while [he’s] taking the test. Instead of [thinking about] video games and those sorts of things*” | |
|  | (…) |  |
| Participant 2.1 | “*But with children, we say, okay, so now we know that you are not a blue flower, you are a pink flower. So we give you pills, so that you can have blue flowers anyway. Well, that is bizarre, right? I think that is completely insane. [Why can’t we] just embrace that this child has pink flowers. It’s great right? It changes things up*”. | |
|  |  |  |
|  | Participant 2.3 | “*Ja, ritalin, dat is de oplossing. Oh ADD, nou, dan krijg je een ritalin, dan uh is ’t goed.”* |
|  | Participant 2.1 | “*nouja, maar goed das geen das geen..”* |
|  | Participant 2.3 | “*Misschien helpt hem wel hoor, dat weet ik niet.”* |
|  | Participant 2.1 | “*Jaja, dat helpt bij mijn zoon heel erg om uh alleen aan zijn toets te denken tijdens zijn toets, in plaats van aan de games en dat soort dingen.”* |
|  |  | (…) |
|  | Participant 2.1 | “*maar van kindjes zeggen we, okay, we weten nu dat jij geen blauwe bloem bent, maar roze bloem, dus we geven jou pillen zodat je toch blauwe bloemen, nou das toch bizar. Ik vind dat echt van de zotten. Omarm, gewoon dat dat kind roze bloemen heeft, leuk toch. Das weer eens wat anders.”* |
|  |  |  |

| ***Theme 5: Adults and Adolescents*** | | |
| --- | --- | --- |
| *Quote Focus group 1: Adults with ADHD*  While introducing herself, one participant mentioned how important her ADHD diagnosis was to her, that a lot had fallen into place when she was classified and that it had given her a much better understanding of herself. When asked what having ADHD said about a person, she said ‘nothing’ and questioned what having ADHD said about her. | | |
|  |  |  |
| Participant 1.2 | “A *lot fell into place for me and I thought, ah, now I understand a lot of things*” | |
|  | (…) |  |
| Moderator | “*What does having ADHD say about a person?*” | |
|  | (…) |  |
| Participant 1.2 | “*Well, my first inkling is [to say] nothing. It actually says nothing. If I look at our society, and that is something I struggle with personally, we always need [to get] a classification first, [ascribe] a label or a box, before someone can get the right help. Because then I wonder, but what does it actually say about me?”* | |
|  |  |  |
|  | Participant 1.2 | “E*r viel voor mij veel op z’n plek, dat ik dacht, ah en nu begrijp ik heel veel dingen”* |
|  |  | (…) |
|  | Moderator | “*Wat zegt het hebben van ADHD over een persoon?*” |
|  |  | (…) |
|  | Participant 1.2 | “*Ja, mijn eerste gevoel is uh niks, eigenlijk zegt het niks ….. uhm, als ik , als ik kijk naar onze maatschappij en dat is wat ik persoonlijk zelf lastig vind, is dat er altijd eerst een classificatie, dus een labeltje of een hokje nodig is voordat iemand de juiste hulp krijg …. Want dan denk ik ja, wat zegt het nou over mij?*” |
|  |  |  |
| *Quote Focus group 3: Clinicians*  One participant first stated that she could not say what ADHD says about a person, because it does not say anything. Yet subsequently, she noted that we diagnose ADHD when people get stuck and that the classification ADHD informs us on brain-functioning, citing the ADHD-brain. | | |
|  |  | |
| Participant 3.0 | “*So, what does it say about a person? I cannot answer that question at all, [it] says absolutely nothing.*” | |
|  | (…) |  |
| Participant 3.0 | “*But we have agreed to diagnose ADHD if someone gets stuck in multiple areas [of life], but you can still have an ADHD-brain. At least that is what I would call it, having an ADHD brain.*” | |
|  |  |  |
|  | Participant 3.0 | “*Dus ja, wat zegt het over een person? Ik kan … Ik kan helemaal die vraag helemaal niet beantwoorden, zegt helemaal niets*” |
|  |  | (…) |
|  | Participant 3.0 | “*Maar we hebben wel afgesproken dat je ADHD vaststelt als iemand vastloopt he, op, op verschillende gebieden, maar je kan nog steeds een een ADHD-brein hebben, ten minste ik zou dat dan wel zo noemen, een ADHD-brein hebben...*” |
|  |  |  |
| *Quote Focus group 2: Parents of children with ADHD*  Similarly, one participant in this group said, within a single sentence, that ADHD means nothing and yet it also means that someone has certain characteristics. | | |
|  |  |  |
| Participant 2.2 | *“Well technically [ADHD means] nothing, no. Certain characteristics, that a label has been attached to.”* | |
|  |  |  |
|  | Participant 2.2 | *“Nou in principe niets, nee. Bepaalde eigenschappen, waar een labeltje aan gehangen is.”* |

**Exemplary quotes for themes specific to (a set of) stakeholder groups**

**Theme 5: Adults and Adolescents with ADHD**

***Quotes Focusgroep 1 – Adults with ADHD***

Participant 1.3: “*Oh yes, I was so happy. And medication, yes, it was like putting on glasses.*”

Participant 1.3: “*Oh ja, ik was zo blij. En medicatie, ja, alsof je een bril opzet zeg.*”

Participant 1.5: “*But, I solved that by saying, society benefits much more if I take Ritalin, and that is why I take it. Not because it makes me better, but because then I can just contribute more. And that is the reason I take it, because otherwise I would also be like, yes, it is actually unfair, but what is unfair about contributing more?*”

Participant 1.5: “*Maar, Ik heb dat opgelost door te zeggen, de maatschappij heeft er veel meer aan als ik Ritalin neem, en daardoor neem ik het, niet omdat ik er zelf beter van word, maar omdat ik dan gewoon meer bij kan dragen. En dat is voor mij de reden om het te nemen, want anders had ik ook zoiets van ja, het is eigenlijk oneerlijk, maar wat is er oneerlijk aan meer bijdragen?*”

Participant 1.1 : “*It is just demonstrably not good for children, you know, growth deficits, but that is why children receive medication, who don’t need it at all, and on the other hand, we deny medication to children medication, who would need it.”*

Participant 1.1 : “*Het is gewoon aantoonbaar niet goed voor kinderen, weetje, groeiachterstanden, maar daardoor krijgen kinderen medicatie, die het helemaal niet nodig hebben, en aan de andere kant krijgen kinderen medi.. he ont.. onthouden medicatie, uhm, die het wel zou nodig zouden hebben*”

***Quotes Focusgroep 7 – Adolescents with ADHD***

Participant 7.2: “*I do have a question, for those of you who take pills, how do you guys feel about those pills?*”

Participant 7.2: “*Ik heb wel een vraag gewoon, uhm, voor degene die de pillen slikken, hoe vinden jullie die pillen?*”

Participant 7.2: “*I always describe it as if there is a Ping-Pong ball in your head, bouncing around, back and forth, and with those pills, that isn’t the case, and I find that pleasant.”*

Participant 7.2: “*Ik beschrijf het altijd als dat er pingpong balletje in je hoofd zo rond, heen en weer gaat, en uhm, met die pillen is dat niet, en dat vind ik wel fijn.*”

Participant 7.4: “*Then I wouldn’t be able to get medication, and I would be all over, it would really be, I don’t know if it would have already happened, but then the whole house would be turned upside down by now.*”

Participant 7.4: “*Dan zou ik geen medicijnen kunnen krijgen en dan zou ik echt overal zijn... dat zou het echt, ik weet of dat al was gebeurd, maar, dan was het hele huis nu afgebroken..”*

Participant 7.1: “*But personally, I liked them, but it was also annoying, you have to do this, otherwise you start to fall behind in school.*”

Participant 7.1: “*Maar zelf, ik vond ze heel fijn, maar het was ook, het was ook vervelend van, je moet je moet dit doen anders weet je kom je kom je kom je eigenlijk niet acht.. loop je achter op school.”*

Participant 7.3: “*That is what I got medication for, but I don’ take it myself, and that is because they make me feel sick.*”

Participant 7.3: “*Daar had ik medicijnen voor gekregen, die slik ik zelf niet, en dat komt omdat ik er zelf uh ziek van word...”*

Participant 7.2: “*And sometimes I try to just pretend that I took my pills, because sometimes I find them a little bit annoying, because then I am suddenly very calm and serious.*”

Participant 7.2: “*En soms probeer ik ook, net alsof te doen, dat ik m’n pillen wel genomen heb, want ik vind ze soms een beetje vervelend, want dan ben ik opeens heel rustig en serieus...*”

**Theme 6: Parents and Teachers**

***Quotes Focusgroep 2 – Parents***

Participant 2.2: “*We have a lot of problems at school, for 3 years now, and actually (they) don’t even know, well, school and the collaborative partners do not know what support he needs’.*

Participant 2.2: “*Wij hebben veel problemen mee op school al uh drie jaar lang, en eigenlijk ook niet eens weten, nouja eigenlijk, school en het samenwerkingsverband weten niet de ondersteuning die hij nodig heeft*”

Participant 2.3: “*You know, he rolls into the system, but no-one really understands it, not even the support person at school.*”

Participant 2.3: “*Weetje, hij zit wel, hij rolt wel in het systeem, maar niemand snapt het echt, zelfs die begeleider op school.”*

Participant 2.3: “*Well, and there (at school) they see, I notice that now, they only see the children for a short while, and they quickly have an impression of ‘well, you don’t really pay attention’, but they don’t really give it much thought.*”

Participant 2.3: “*Nou en daar zien ze, dat merk ik nu, ze zien de kinderen maar kort, en ze hebben al snel een beeld erbij van ‘nouja, jij let niet zo goed op’, besteden er niet zo heel veel aandacht aan.”*

Participant 2.1: “*I really, not once, but on multiple occasions, left the school crying because I couldn’t get through to them.*

Participant 2.2: “*Very frustrating, that powerlessness..*”

Participant 2.1: “*Ik ben echt, niet een keer, meerdere keren huilend de school uitgelopen, omdat ik er niet doorheen kwam.”*

Participant 2.2: “*Heel frustrerend, die machteloosheid..*”

Participant 2.1: “*No, but it has to do, I think, and I will say it out loud, with the arrogance of the teacher, who thinks that you (as a parent) don’t really see them (the children). I find that so presumptuous.*”

Participant 2.1: “*Nee, maar het heeft ook te maken vind ik, met en dan ik noem het even echt hardop, met de arrogantie van de leerkracht, die denkt dat jij hen niet goed ziet … Dat vind ik echt zo aanmatigend.”*

Participant 2.3: “*In fifth grade, we unfortunately ran into a teacher, with whom it didn’t click, and then it all went wrong. Then they also said we should get him tested. And Ritalin was mentioned immediately, and we weren’t comfortable with that at the time.*”

Participant 2.3: “*Groep 5 helaas tegen een juf aangelopen met wie het niet klikte, en toen ging het helemaal mis, toen werd er ook geroepen van zou je hem niet eens laten testen. En al gelijk werd er ook Ritalin genoemd, en, dat zagen wij op dat moment niet zitten.*”

**Quotes Focusgroep 4 – Teachers**

Participant 4.6: “*But there is never anything wrong with the child, it is always the teacher, in my opinion.*”

Participant 4.6: “*Maar er ligt echt nooit wat aan het kind, het ligt altijd aan de docent, ben ik van mening..”*

Participant 4.2: “*But it is all about money, it is all about getting that piece of paper (diploma) and how a student gets from A to B doesn’t really matter to them (the schools) at all.*”

Participant 4.2: “*Maar het draait allemaal om geld, het draait allemaal maar om het papiertje binnen halen en hoe de leerling van A naar B gaat dat boeit ze eigenlijk helemaal niet*.”

Participant 4.6: “*We are not on the right track at all. We are putting students into a cell, and we call that a classroom, and they have to sit there and listen to us...*”.

Participant 4.6: “*Wij zijn niet goed bezig hoor, wij stoppen leerlingen in een hok, dat heet een klaslokaal daar moeten ze zitten, en dan moeten ze luisteren naar ons.*”

Participant 4.1: “*Space, we just need more money, because our curriculums are so full, there is an enormous workload, and I would love to be a certain kind of teacher, but I am definitely not always that teacher.*”

Participant 4.1: “*Ruimte, je hebt gewoon meer geld nodig, want onze onze uh lesprogramma zitten zo vol, der is enorme werkdruk, dus ik zou uh, ik zou supergraag uh uh een bepaalde docent willen zijn, maar die ben ik lang niet altijd.*”

**Theme 7: Researchers**

Participant 5.5: “*Yes, but I think indeed that it is a disadvantage that people sort of see it as, oh I have ADHD, so it is because of that... Then you start to see it as an explanation, which it isn’t really, of course, because it is actually more of a description of how a child behaves..*”

Participant 5.5: “*Ja, maar ik denk dat dat inderdaad wel het nadeel is dat mensen dan het als een soort van, oh ik heb ADHD, dan komt het dus daardoor... Dat je dat dan als een soort verklaring wordt gezien, wat het eigenlijk natuurlijk niet echt is, omdat het eigenlijk gewoon meer een beschrijving is van hoe een kind zich gedraagt…”*

Participant 5.6: “*Well, I think that researchers themselves have slowly started to believe that it (ADHD) is a concrete thing... that is the thing about these terms, if they exist for a long time, they start to live a life of their own. And then that makes me think about my own neuroscientific research and many of you have also done this. On some level think that it also secretly plays a role in my thinking, that I make it more of a thing (ADHD) than it really is.”*

Participant 5.6: “*Nouja, ik denk dat onderzoekers zelf ook dus langzaam zijn gaan geloven in dat het een heel concreet ding is.. dat is gewoon met die termen, als die lang bestaan, dan gaan ze echt een leven op zichzelf vormen. En ik moet dan ook een beetje denken aan mijn eigen neurowetenschappelijke onderzoek en hebben veel van jullie ook gedaan. Ergens denk ik dat het stiekem bij mij toch ook soms een rol speelt, dat ik het toch wat meer tot een ding maak dan dat het werkelijk is.*”

Participant 5.6: “*I think that if we all collectively use that word (ADHD), then it becomes much more of a thing. So, then I go back to the term reification, and I think that can all have consequences for how such a disorder, such a label, is interpreted by a child.”*

Participant 5.6: “*Ik denk als we allemaal massaal dat woord gebruiken, dat het wel meer een tot ding wordt gemaakt, dus ik pak dan toch dat woord reificatie erbij, en dat kan denk ik wel allemaal gevolgen hebben over hoe zo’n stoornis, hoe zo’n label bij een kind overkomt.*”

Participant 5.6: “*I also developed a lot of affinity for today’s topic, because I suspect that the we talk about ADHD affects how children think about themselves and (their) autonomy, and eventually, how that affects mindset and treatment success, so I have a lot of interest in that.”*

Participant 5.6: “*Ik heb ook wel affiniteit gekregen uh met het onderwerp van vandaag ook doordat ik uh, ja, wel vermoedens heb dat uh de manier waarop we over ADHD spreken dat dat uh effecten heeft op uh, kinderen hoe ze denken over zichzelf en autonomie en hoe dat uiteindelijk effect heeft op mindset en behandelsucces, dus ik heb daar heel veel interesse naar.*”

Participant 5.5: “*A person has ADHD, so then if it is ADHD, is it more like part of who you are, and if you say depression, then it is more like, I have this now, but it might pass, maybe?”*

Participant 5.5: “*Diegene heeft een ADHD, dus dan als het ADHD is, is het meer alsof het een soort van onderdeel is van wie je bent, en als je zegt een depressie dan is het meer van ja ik heb dit nu, maar het gaat ook wel weer over, misschien?*”
