# Supplementary material for: Hidden in plain sight: how individual ADHD stakeholders have conflicting ideas about ADHD but do not address their own ambivalence
Source: Eur Child Adolesc Psychiatry. 2023 Sep 9;33(6):1921–33. doi: 10.1007/s00787-023-02290-w (PMC11211115; doi:10.1007/s00787-023-02290-w)
Supplement: Supplementary file 4 — Supplementary file4 (DOCX 54 KB) [file 787_2023_2290_MOESM4_ESM.docx]

Supplement 4: Overview and translation of axial coding schemes for focus group analyses

**Focus group 1: Adults**

| - ADHD as an identity   - ADHD is what I am   - Own understanding and interpretation of ADHD   - Use of labels as identity markers at home - ADHD is shit and cool too - ADHD stems from societal and internalized norms   - ADHD as an indication that you fall outside the norm   - The need for labels has increased in society   - Holding yourself accountable if you cannot meet the standard   - Not conforming to the norm can lead to stigmatization   - Expressing doubts about social norms   - Wanting a world in which everyone can shape their own environment. - ADHD says something about a person   - ADHD indicates that things can quickly go wrong   - ADHD indicates that someone is different   - ADHD indicates that someone needs extra support   - ADHD indicates that you are more troubled by certain problems   - ADHD is a base of symptoms (despite great variability)   - ADHD can express itself at inappropriate times   - ADHD explained behaviour   - ADHD does not necessarily say everything about abnormalities   - Clarity of the presence of ADHD   - Explanation based on your own interpretation sometimes works - ADHD does not say about a person   - ADHD is too restrictive in describing a person   - ADHD is too simplified and arbitrary   - ADHD is variable; manifests itself differently in everyone   - ADHD says little, but is necessary for care and support   - Emphasizing that someone is different and has their own characteristics   - Much is unclear about ADHD   - Focus away from the label and on the environment and situation   - Doubts about what ADHD means or entails - The happy person with ADHD   - Difficult to get a diagnosis, but only happy with it yourself   - Finding recognition in diagnosis; satisfied and happy   - Celebrating getting the diagnosis - This focus group is only part of the story - Medication   - Describe positive effects of medication   - Self-doubt about taking medication   - There should be more clarity and knowledge about medication   - Uses medication, despite own resistance   - Medication is necessary, because lifestyle has been adjusted   - Taking medication to achieve a goal   - Curiosity about the effects of medication   - Stopped despite usefulness of medication   - Caution with medication for children. - People with ADHD have a brain that works differently   - ADHD has to do with connecting and disconnecting attention   - People with ADHD react differently to feedback   - People with ADHD are better at associating - Reasons for seeking a diagnosis   - Have ADHD examined because it was no longer possible   - Recognition in experiences of family and environment   - Diagnosis due to suggestion of care provider   - Seek the possibility of appropriate assistance   - Looking for an explanation why things don't work out   - Late diagnosis due to own compensation - Connection with other disorders   - Rejection of other labels; they are not useful   - Other labels instead of or in addition to ADHD   - Much overlap and comorbidity with other disorders   - Responsibility research to find associations. - Stigmatizing effect of the ADHD label   - Fear of consequences at work   - Feeling limited by prejudices   - With ADHD you are judged on your exterior   - Being dismissed as incapable and unreliable - What does the label ADHD do (benefits)   - Brings about understanding of problems   - Provides guidance when learning to deal with limitations   - Provides guidance and clarity   - Indicates how someone should be treated   - Is necessary for the distribution of care   - Explains why things don't work out   - Induce a feeling of liberation   - Makes people think for themselves - What the label ADHD does not do (disadvantages)   - Leads to fear of stigma (not wanting to tell others)   - Does not take away difficulties   - Focus on the negative   - Suggests that people with ADHD all need the same thing   - Often lack of recognition   - Loses meaning due to vagueness and lack of recognition   - Causes less acceptance for variation (boxing)   - Causes doubt about measurability of diagnoses - What should we do as a society with ADHD   - As a society we cannot do without labels   - Importance of counteracting the stereotype   - The media plays an important role in providing information   - There is too little knowledge about ADHD   - People with lived experience should take more control   - Everyone should be able to shape their own environment   - Letting go of opinions of others   - Need more education about ADHD   - Don't just focus on things that don't work out   - Sensationalizing and polarization work against knowledge   - ADHD stigma is changing   - The term ADHD is used too loosely   - Expectation that labels will no longer be needed at some point - What should we do in healthcare for ADHD   - ADHD is not very meaningful, but is necessary for care   - Importance of offering multiple options for care   - When explaining ADHD, often a lack of nuance and contextualization   - Too little proper support is given   - Experience that care providers often do not know enough about ADHD   - Focus can be directed more towards the positive   - Caregiver can often take on a more listening role   - You must also take some responsibility yourself   - Support and information for people with ADHD is very much lacking   - Expressing the usefulness of individually tailored assistance - Word choice ADHD   - Need for new terminology   - Be aware of how you talk about your own ADHD   - Disliking the term ADHD   - Despite disadvantages, term ADHD is needed   - Tension between terminology disorder and abnormality   - Terminology sometimes sensitive due to implications | - ADHD als identiteit   - ADHD is wat ik ben   - Eigen begrip en interpretatie van ADHD   - Gebruikt van labels als identiteitsmarkers thuis - ADHD is shit en ook gaaf - ADHD komt voort uit de maatschappelijke en geïnternaliseerde normen   - ADHD als indicatie dat je buiten de norm valt   - Behoefte aan labels is in de samenleving groter geworden   - Jezelf erop afrekenen als je niet aan de norm kunt voldoen   - Niet conformeren aan de norm kan leiden tot stigmatisering   - Uitdrukken twijfels over maatschappelijke normen   - Het willen van een wereld waarin iedereen zijn eigen omgeving kan vormgeven. - ADHD zegt iets over een persoon   - ADHD geeft aan dat het snel verkeerd kan gaan   - ADHD geeft aan dat iemand anders is   - ADHD geeft aan dat iemand extra ondersteuning nodig heeft   - ADHD geeft aan dat je meer last hebt van bepaalde problemen   - ADHD is een basis van symptomen (ondanks grote variabiliteit)   - ADHD kan zich op ongepaste momenten uitten   - ADHD verklaard gedrag   - ADHD zegt niet per se alles over afwijkingen   - Duidelijkheid van de aanwezigheid van ADHD   - Uitleg aan de hand eigen interpretatie werkt soms - ADHD zegt niet over een persoon   - ADHD is te beperkend in de omschrijving van een persoon   - ADHD is te versimpeld en arbitrair   - ADHD is variabel; uit zich bij iedereen verschillend   - ADHD zegt weinig, maar is nodig voor hulpverlening   - Benadrukken dat iemand anders is en zijn eigen kenmerken heeft   - Er is veel onduidelijk over ADHD   - Focus weg van het label en op omgeving en situatie   - Twijfel over wat ADHD betekent of inhoudt - De blije ADHD-er   - Moeilijk om een diagnose te krijgen, maar zelf alleen blij zijn ermee   - Erkenning vinden in diagnose; tevreden en blij   - Vieren van het krijgen van de diagnose - Deze focusgroep is slechts een deel van het verhaal - Medicatie   - Beschrijven positieve effecten medicatie   - Eigen twijfel over nemen medicatie   - Er moet meer duidelijkheid en kennis komen over medicatie   - Gebruikt medicatie, ondanks eigen weerstand   - Medicatie is nodig, want leefwijze aangepast   - Nemen medicatie om een doel te bereiken   - Nieuwsgierigheid naar de effecten van medicatie   - Ondanks nut medicatie, toch gestopt   - Voorzichtigheid bij medicatie voor kinderen. - Mensen met ADHD hebben een brein dat anders werkt   - ADHD heeft te maken met koppelen en loskoppelen van aandacht   - Mensen met ADHD reageren anders op feedback   - Mensen met ADHD zijn beter in associëren - Redenen voor het zoeken naar een diagnose   - ADHD laten onderzoeken omdat het niet meer ging   - Erkenning in ervaringen familie en omgeving   - Diagnose vanwege suggestie hulpverlener   - Mogelijkheid tot juiste hulpverlening zoeken   - Zoeken naar een verklaring waarom dingen niet lukken   - Late diagnose vanwege compensatievermogen - Samenhang met andere stoornissen   - Afwijzing andere labels; die zijn niet nuttig   - Andere labels in plaats van of naast ADHD   - Veel overlap en comorbiditeit met andere stoornissen   - Verantwoordelijkheid onderzoek om associaties te vinden. - Stigmatiserende werking van het label ADHD   - Angst voor consequenties op werk   - Beperkt voelen door vooroordelen   - Bij ADHD word je afgerekend op je buitenkant   - Weggezet worden als incapabel en onbetrouwbaar - Wat doet het label ADHD (voordelen)   - Bewerkstelligt begrip voor problemen   - Geeft houvast bij het leren omgaan met beperkingen   - Geeft houvast en duidelijkheid   - Indiceert hoe iemand moet worden behandeld   - Is nodig voor verdeling van zorg   - Verklaart waarom dingen niet lukken   - Veroorzaak gevoel van bevrijding   - Zorgt dat mensen uit hun eigen kracht gaan denken - Wat doet het label ADHD niet (nadelen)   - Leidt tot angst voor stigma (andere niet willen vertellen)   - Neemt moeilijkheden niet weg   - Richt de focus op het negatieve   - Suggereert dat ADHD-ers allemaal hetzelfde nodig hebben   - Vaak gebrek aan herkenning   - Verliest betekenis door vaagheid en gebrek aan herkenning   - Veroorzaak minder acceptatie voor variatie (hokjes plaatsen)   - Veroorzaak twijfel over meetbaarheid diagnoses - Wat moeten we als maatschappij met ADHD   - Als samenleving kunnen we niet zonder labels   - Belang van het tegengaan van het stereotype beeld   - De media speelt een belangrijke rol bij voorlichting   - Er is te weinig kennis over ADHD   - Ervaringsdeskundigen zelf moeten meer regie nemen   - Iedereen zou zijn eigen omgeving moeten kunnen vormgeven   - Loslaten van meningen anderen   - Meer educatie over ADHD benodigd   - Richt de focus niet alleen op dingen die niet lukken   - Sensationalisering en polarisering werkt kennis tegen   - Stigma ADHD is aan het veranderen   - Term ADHD wordt te losjes gebruikt   - Verwachting dat op een gegeven moment labels niet meer nodig zijn - Wat moeten we in de hulpverlening met ADHD   - ADHD zegt weinig, maar is nodig voor hulpverlening   - Belang van aanbieden meerdere opties hulpverlening   - Bij uitleg ADHD, vaak gebrek aan nuancering en contextualisatie   - Er wordt te weinig goede begeleiding gegeven   - Ervaring dat hulpverleners ook vaak niet goed weten wat ADHD is   - Focus kan meer worden gericht op het positieve   - Hulpverlener kan vaak een meer luisterende rol innemen   - Je moet ook voor een deel zelf verantwoordelijkheid nemen   - Ondersteuning en voorlichting voor mensen met ADHD mist heel erg   - Uitdrukken van nut individueel afstemde hulpverlening - Woordkeuze ADHD   - Behoefte aan nieuwe terminologie   - Bewust zijn hoe je over eigen ADHD praat   - Hekel hebben aan de term ADHD   - Ondanks nadelen, is term ADHD nodig   - Spanningsveld tussen stoornis en afwijking   - Woordkeuze soms gevoelig door implicaties |
| --- | --- |

**Focus group 2: Parents**

| - School   - Problems at school central to diagnosis   - Where things go wrong in the school system   - Label due to expectations and pressure of the school   - Importance and quality of teacher and school   - Personal knowledge of children is important - Medication   - Importance and utility   - Limitation of human variation   - Side effects   - Effectiveness   - Child needs help remembering   - Child develops resistance, wants to discover own identity - Label ADHD   - Reasons for diagnosis   - How do parents themselves use the label?   - A label says about a child…   - A label has a positive effect   - A label has a negative effect   - Errors in the use of the ADHD label   - Link between ADHD and other labels - Experiences of parents   - Importance of upbringing and parental involvement   - Parents rely on their own knowledge and instinct   - As parents you have to be strong   - Parents need outside support and tips   - Parents looking for tailor-made solutions for their child   - Lack of guidance for parents   - Parents find recognition in each other   - Importance of changing things from above - How to deal with ADHD   - Acceptance of child characteristics   - More customization for children   - More awareness and knowledge about ADHD   - More support for children   - Label is not an excuse   - Child must always be the focal point   - Changes in the system - Noticeable phrasing | - School   - Schoolproblemen centraal aan diagnose   - Waar het mis gaat in het schoolsysteem   - Label door verwachting en druk school   - Belang en kwaliteit van leerkracht en school   - Persoonlijke kennis van kinderen belangrijk - Medicatie   - Belang en nut   - Beperking van menselijke variatie   - Bijwerkingen   - Effectiviteit   - Helpen herinneren kind   - Ontwikkeling weerstand en eigen identiteit ontdekken - Label ADHD   - Redenen voor diagnose   - Hoe gebruiken ouders zelf het label   - Een label zegt over een kind…   - Een label heeft een positief effect   - Een label heeft een negatief effect   - Fouten in het gebruik van het label ADHD   - Link tussen ADHD en andere labels - Ervaringen van ouders   - Belang van opvoeding en betrokkenheid ouder   - Ouders vertrouwen op eigen kennis en instinct   - Als ouders moet je sterk in je schoenen staan   - Behoefte ouders aan ondersteuning en tips buitenaf   - Ouders op zoek naar maatwerk voor kind   - Gebrek aan begeleiding voor ouders   - Ouders vinden erkenning bij elkaar   - Belang om van bovenaf dingen te veranderen - Hoe om te gaan met ADHD   - Acceptatie van kenmerken kind   - Meer maatwerk voor kinderen   - Meer bewustzijn en kennis over ADHD   - Meer ondersteuning voor kinderen   - Label is geen excuus   - Kind moet centraal staan   - Veranderingen in het systeem - Bijzondere verwoordingen |
| --- | --- |

**Focus group 3: Clinicians**

| - What is ADHD   - ADHD says nothing   - ADHD indicates that not everything is going well   - ADHD is falling outside of the norm   - ADHD is a manual   - Distinguishing between different diagnoses   - ADHD does not always go away   - Family recognition ADHD   - The diagnosis is for…   - ADHD with or without dysfunction   - Not fitting in the normal context   - Vagueness ADHD   - Difference in diagnosis between boys and girls   - Question about origin of ADHD - Importance of the brain   - Understanding of the brain is important   - Understanding of the brain not important   - Complexity brain functioning   - Biological explanation in psychoeducation   - The ADHD brain - What is the importance of the diagnosis   - Positive effects diagnosis   - Negative effects diagnosis   - Why are there diagnoses   - Doubts about the value of the diagnosis   - Diagnosis footnotes - Focus on the individual   - ADHD becomes part of the identity   - Adjustments by the environment are important   - Tune in to the child   - Importance of environmental response   - Importance of personal experience   - Importance of focusing on positive qualities ADHD   - Importance of accepting yourself   - Importance of thinking about what ADHD means   - Involving the individual in the discussion – agency   - Putting the person ahead of the diagnosis   - Focus on dynamic aspects of development   - Everyone has pluses and minuses   - Individual differences in ADHD diagnosis - Responsibility of clinicians   - Perspective of clinicians versus parents   - Good use of diagnoses and DSM   - Clinicians part of societal debate   - Providing correct information and perspective to the client   - Clinical experience of great importance - Discussion DSM system   - Doubts about the system   - Discussion complicated and polarizing   - DSM also provides guidance   - No need for DSM classification   - None of the disorders in the DSM are real   - Criticism of mental health care for youth   - Critical discussion about ADHD   - Label is quickly given when deviating from the norm   - Nothing wrong with classifying   - Recently started to critically look at the DSM   - Doubts about the sustainability of the concepts   - Change of care system required - Wording   - ADHD as a swearword, not as a diagnosis   - Diagnosis is a classification   - Health care workers do not label   - Inflation of classification   - Difficulty word 'Label'   - Societal impact on diagnosis and psychiatry   - Switching to another term - Interaction between society and diagnosis   - As a society, becoming more lenient towards the behavior of children   - Diagnosis as an excuse for not excelling   - Discussion on ADHD being dependent on context   - There should be more room for the development of children   - Functioning largely dependent on adaptation by environment   - Environment and parents impact a child’s understanding   - You get ADHD by not being approached properly   - Societal expectations of children need to be adjusted   - Dealing with ADHD is very dependent on environment   - Excessive societal expectations vs. psychiatry   - Change in brain development by society   - What is normal functioning and what is not - What improvements are needed   - Teachers should have more knowledge of development   - More representatives for children needed   - Negative press and perspective on ADHD   - Education not designed by knowledgeable people   - Role models and guidance for children   - Stereotypes in the media complicate the diagnosis | - Wat is ADHD   - ADHD zegt niets   - ADHD geeft aan dat het niet helemaal goed loopt   - ADHD is vallen in buitencategorie   - ADHD is een gebruiksaanwijzing   - Onderscheid maken verschillende diagnoses   - ADHD gaat lang niet altijd over   - Familie-herkenning ADHD   - De diagnose is voor…   - ADHD met of zonder disfunctioneren   - Niet passen in de normale context   - Vaagheid ADHD   - Verschil diagnose tussen jongens en meisjes   - Vraag over origine ADHD - Belang van het brein   - Begrip van het brein wel belangrijk   - Begrip van het brein niet belangrijk   - Complexiteit werking brein   - Biologische uitleg in psychoeducatie   - Het ADHD-brein - Wat is het belang van de diagnose   - Positieve effecten diagnose   - Negatieve effecten diagnose   - Waarom zijn er diagnoses   - Twijfels over de waarde van de diagnose   - Voetnoten bij diagnose - Focus op het individu   - ADHD wordt onderdeel van de identiteit   - Afstemmen omgeving van groot belang   - Afstemmen op het kind   - Belang van reactie van omgeving   - Belang van ervaring van de persoon zelf   - Belang van focus op positieve kwaliteiten ADHD   - Belang van jezelf accepteren   - Belang van nadenken over wat ADHD betekent   - Betrekken individu bij discussie – agency   - Eerst de persoon voor de diagnose   - Focus op dynamische aspecten ontwikkeling   - Iedereen heeft plussen en minnen   - Individuele verschillen ADHD-diagnose - Verantwoordelijk van clinici   - Perspectief clinici versus ouders   - Goede inzet van diagnoses en DSM   - Clinici onderdeel van maatschappelijk debat   - Juiste informatie en perspectief meegeven aan client   - Klinische ervaring van groot belang - Discussie DSM-systeem   - Twijfels over het systeem   - Discussie ingewikkeld en polariserend   - DSM geeft ook houvast   - Geen behoefte aan stellen DSM-classificatie   - Geen van de stoornissen in de DSM is echt   - Kritiek op jeugd-zorgsysteem   - Kritische discussie rondom ADHD   - Label wordt snel gegeven bij afwijking norm   - Niets mis met classificeren   - Recent kritisch naar DSM gaan kijken   - Twijfel over houdbaarheid van de concepten   - Verandering zorgsysteem nodig - Verwoording   - ADHD als scheldwoord, niet als diagnose   - Diagnose is een classificatie   - Hulpverleners labelen niet   - Inflatie van classificatie   - Lastigheid woord ‘Label’   - Maatschappelijke werking op diagnose en psychiatrie   - Wisselen naar een andere term - Wisselwerking maatschappij en diagnose   - Als maatschappij coulanter worden naar gedrag kinderen   - Diagnose als excuus voor niet excelleren   - Discussie over contextafhankelijkheid ADHD   - Er moet meer ruimte komen voor ontwikkeling van kinderen   - Functioneren grotendeels afhankelijk van aanpassing omgeving   - Impact van omgeving en ouders op begrip kind   - Krijg je ADHD door niet goed benadert te worden   - Maatschappelijke verwachtingen van kinderen moeten worden aangepast   - Omgang met ADHD heel afhankelijk van omgeving   - Te grote maatschappelijke verwachtingen vs. psychiatrie   - Verandering hersenontwikkeling door maatschappij   - Wat is normaal functioneren en wat niet - Welke verbeteringen zijn nodig   - Leerkrachten moeten meer kennis hebben van ontwikkeling   - Meer vertegenwoordigers van kinderen nodig   - Negatieve pers en perspectief op ADHD   - Onderwijs niet vormgegeven door mensen die er verstand van hebben   - Rolmodellen en houvast voor kinderen   - Stereotiep media vermoeilijkt diagnostisering |
| --- | --- |

**Focus group 4: Teachers**

| - What does ADHD say   - ADHD is binary; it is present or not   - ADHD (often) says something about the upbringing   - ADHD says something about behavior   - ADHD says something about the need for support   - ADHD says nothing about the person   - Diagnosis important for whether or not to give medication   - Diagnosis says child can't help it - Advantages of diagnosis   - Diagnosis gives direction   - Diagnosis gives peace of mind   - Diagnosis provides a quick overview of the situation   - Diagnosis leads to more patience   - Diagnosis leads to more understanding   - Diagnosis leads to focus on educational needs   - Diagnosis takes blame away   - Diagnosis opens doors for help   - Diagnosis provides an explanation   - Diagnosis is experienced as helpful - Disadvantages of diagnosis   - Diagnosis confirms the negative   - Diagnosis used by parents as an excuse   - Diagnosis can experienced as traumatic   - Diagnosis closes doors and hinders young people   - Diagnosis too generalizing   - Diagnosis has a restrictive effect, tunnel vision   - Diagnosis is used as an excuse   - No support without classification   - Children can behave according to label   - Labeled children see themselves as different   - Medication often seen at the goal, instead of a means   - Doubts about arbitrary diagnoses   - Comments on diagnosis - Individual vs. Diagnosis   - Importance of getting to know the individual   - Importance of looking at needs for support   - Importance of looking at solutions for the child   - Behavior more important than diagnosis   - N=1   - Sometimes you have to look past the label   - Variation within ADHD diagnosis - What is going wrong in the education system   - Adjustments in schools necessary   - Limited adaptability in education   - If education fails, it is often blamed on the student   - Coaching of teachers is minimal   - Importance of open communication about diagnoses   - Important role of money   - Often only the content of classes is pushed   - Teachers and education often boring and bad   - Mismatch between the different interests   - Traditional education is maintained   - Workload can also make adjustments difficult - Interaction and possible improvements in education   - Agency of the child   - Responsibility of teachers   - Changes in the system   - Communication and relationship with child   - Diagnosis not discussed in class   - More understanding in education than in society   - In special education more room for adaptation to the child   - Quality education of great importance - Improvements in using diagnosis   - Children should not use diagnosis   - Inform and instruct children properly about the use of diagnosis   - Parents must also be able to recognize the needs of children | - Wat zegt ADHD   - ADHD is binair; het is wel of niet aanwezig   - ADHD zegt (vaak) iets over de opvoeding   - ADHD zegt iets over gedrag   - ADHD zegt iets over ondersteuningsbehoefte   - ADHD zegt niets over de persoon   - Diagnose belangrijk voor wel of niet geven medicatie   - Diagnose zegt dat kind er niets aan kan doen - Voordelen van diagnose   - Diagnose geeft richting   - Diagnose geeft rust   - Diagnose geeft snel overzicht van situatie   - Diagnose leidt tot meer geduld   - Diagnose leidt tot meer begrip   - Diagnose leidt tot focus op onderwijsbehoeften   - Diagnose onschuldigt   - Diagnose opent deuren voor hulp   - Diagnose werkt verklarend   - Diagnose wordt als helpend ervaren - Nadelen van diagnose   - Diagnose bevestigt het negatieve   - Diagnose door ouders gebruikt als ontschuldiging   - Diagnose kan traumatisch werken of zijn   - Diagnose sluit deuren en belemmert jongeren   - Diagnose te generaliserend   - Diagnose werkt beperkend, tunnelvisie   - Diagnose wordt als excuus gebruikt   - Geen ondersteuning zonder classificatie   - Kinderen kunnen zich gaan gedrag naar label   - Kinderen met label zien zichzelf als anders   - Medicatie vaak doel in plaats van middel   - Twijfel rondom willekeur diagnoses   - Kanttekeningen bij diagnose - Individu vs. Diagnose   - Belang van het individu leren kennen   - Belang van kijken naar ondersteuningsbehoefte   - Belang van kijken naar oplossingen voor kind   - Gedrag belangrijker dan diagnose   - N=1   - Soms moet je voorbij het label kijken   - Variatie binnen ADHD-diagnose - Wat gaat er mis in het onderwijs   - Aanpassingen school benodigd   - Aanpassingsvermogen in het onderwijs beperkt   - Als leren niet lukt, wordt het vaak bij de leerling gelegd   - Begeleiden van docenten gebeurt minimaal   - Belang van open communicatie over diagnoses   - Belangrijke rol van geld   - Er wordt vaak alleen gedramd op de inhoud   - Leerkrachten en onderwijs vaak saai en slecht   - Mismatch tussen de verschillende belangen   - Traditioneel onderwijs wordt in stand gehouden   - Werkdruk kan aanpassingen ook lastig maken - Omgang en mogelijke verbeteringen in het onderwijs   - Agency van het kind   - Verantwoordelijkheid docenten   - Veranderingen systeem   - Communicatie en relatie met kind   - Diagnose niet klassikaal besproken   - In onderwijs relatief veel begrip i.v.m. maatschappij   - In speciaal onderwijs meer ruimte voor aanpassen aan kind   - Kwaliteit onderwijs van groot belang - Verbeteringen gebruik diagnose   - Kinderen moeten diagnose niet inzetten   - Kinderen goed informeren en instrueren over gebruik diagnose   - Ouders moet opvoedingsbehoeften van kinderen ook kunnen herkennen |
| --- | --- |

**Focus group 5: Researchers**

| - What does having ADHD mean   - ADHD means there is a problem   - ADHD means someone meets the criteria   - ADHD means someone has sought help   - ADHD means that someone is experiencing symptoms   - ADHD means that someone is stuck and limited   - ADHD means that someone has been for diagnostic testing   - ADHD is not necessarily chronic   - ADHD is often seen as chronic   - ADHD does not say everything about a person   - The individual in ADHD   - Not all symptoms need to be present for a diagnosis - Why the diagnosis of ADHD is sought after   - ADHD is often seen as an explanation   - People like to think in boxes   - Parents often seek diagnosis   - System of psychiatry is built on diagnoses   - Word ADHD important for communication and research - ADHD classification has positive effects   - ADHD classification can spare self-esteem   - ADHD classification leads to relief   - ADHD classification takes away blame   - ADHD can turn out more positively in older age   - ADHD ensures clearer communication   - Goal ADHD is to help people - ADHD classification has negative effects   - ADHD classification often takes its own course in people's heads   - ADHD classification can lead to thinking in terms of limitations   - ADHD classification is sometimes received with grief (chronicity)   - Reality of use of ADHD deviates from the ideal   - Sometimes parents do not want a diagnosis   - The search for diagnosis is sometimes valued over getting help   - Comparison of medical and psychiatric model - The biology of ADHD   - ADHD runs in families   - ADHD often explained biologically (brain)   - Biology cannot help clinical practice any further   - Interactions biology and environment are complex   - Importance of emphasizing brain plasticity   - Neurobiological examination - Psychoeducation and explanation of ADHD   - Biological explanation that influences the mindset   - Communication with parents difficult without the word ADHD   - Contact with peers is important in psychoeducation   - Medication must be properly embedded in care   - Spending a lot of time with parents to explain ADHD   - Parents often focus on the classification in the letter   - Psychoeducation aims to strengthen self-image. - Discussion debate ADHD   - Debate indicates change in psychiatry   - Negative note in the debate   - Curiosity about conversation ADHD   - Researchers understand the nuances of ADHD well   - This discussion is also relatively new for researchers   - Pointing out conflict   - Thinking a lot about the topic of focus group - Terminology in the debate   - How do we use terminology at the moment   - Opinions 'classification', 'diagnosis' and 'label;   - Difficulties in Terminology   - Reification takes place through terminology   - Possible changes in language use   - Difference between depression and ADHD in wording - Alternative ADHD   - Dimensional thinking   - Difficult to imagine a world without diagnoses   - Creating subgroups of ADHD   - Network analysis   - Stratification based on treatment outcomes - Improvements in how to deal with ADHD   - Improvements in in treatment   - Improvements in Society   - Improvements in Research | - Wat betekent het hebben van ADHD   - ADHD betekent dat er een probleem is   - ADHD betekent dat iemand aan de criteria voldoet   - ADHD betekent dat iemand hulp heeft gezocht   - ADHD betekent dat iemand tegen symptomen aanloopt   - ADHD betekent dat iemand vastloopt en beperkt is   - ADHD betekent dat iemand voor diagnostisch onderzoek is geweest   - ADHD is niet per definitie chronisch   - ADHD wordt vaak gezien als chronisch   - ADHD zegt niet alles over een persoon   - Het individu in ADHD   - Voor diagnose hoeven niet alle symptomen aanwezig te zijn - Waarom de diagnose ADHD gewild is   - ADHD wordt vaak gezien als verklaring   - Mensen denk graag in hokjes   - Ouders vaak op zoek naar diagnose   - Systeem psychiatrie is ingericht op diagnoses   - Woord ADHD belangrijk voor communicatie en onderzoek - ADHD-classificatie heeft positieve uitwerkingen   - ADHD-classificatie kan het zelfbeeld sparen   - ADHD-classificatie leidt tot opluchting   - ADHD-classificatie werkt ontschuldigend   - ADHD kan op oudere leeftijd positiever uitpakken   - ADHD zorgt voor duidelijkere communicatie   - Doel ADHD is het helpen van mensen - ADHD-classificatie heeft negatieve uitwerkingen   - ADHD-classificatie gaat vaak eigen leiden in het hoofd van mensen   - ADHD-classificatie kan leiden tot denken in beperkingen   - ADHD-classificatie wordt soms met rouw ontvangen (chroniciteit)   - Realiteit gebruik ADHD wijkt af van het ideaal   - Soms willen ouders geen diagnose   - Zoektocht diagnose soms belangrijker dan hulpvraag   - Vergelijking medisch en psychiatrisch model - De biologie van ADHD   - ADHD in de familie   - ADHD vaak biologisch uitgelegd (brein)   - Biologie kan kliniek en praktijk niet verder helpen   - Interacties biologie en omgeving zijn complex   - Nadruk op plasticiteit hersenen van belang   - Neurobiologisch onderzoek - Psychoeducatie en uitleg van ADHD   - Biologische uitleg die invloed heeft op de mindset   - Communicatie ouders lastig zonder woord ADHD   - Lotgenoten contact belangrijk bij psychoeducatie   - Medicatie moet goed worden ingebed in zorg   - Met ouders veel tijd besteden aan uitleg ADHD   - Ouders focussen vaak op de classificatie in de brief   - Psychoeducatie heeft bedoeling het zelfbeeld te versterken. - Bespreking debat ADHD   - Debat indiceert verandering in psychiatrie   - Negatieve noot in het debat   - Nieuwsgierigheid naar gesprek ADHD   - Onderzoekers begrijpen nuances ADHD goed   - Ook voor onderzoekers is deze discussie relatief nieuw   - Tweestrijd benoemd   - Veel nagedacht over gespreksonderwerp - Terminologie in het debat   - Hoe gebruiken we terminologie op dit moment   - Meningen ‘classificatie’, ‘diagnose’ en ‘label;   - Moeilijkheden terminologie   - Door terminologie vindt reïficatie plaats   - Mogelijk veranderingen in taalgebruik   - Verschil depressie en ADHD in bewoording - Alternatieve ADHD   - Dimensie-denken   - Lastig om een wereld zonder diagnoses voor te stellen   - Maken subgroepen ADHD   - Netwerk-analyse   - Stratificeren op basis van behandelresultaten - Verbeteringen in omgang ADHD   - Verbeteringen in behandeling   - Verbeteringen in Maatschappij   - Verbeteringen in Onderzoek |
| --- | --- |

**Focus group 6: Policy Makers**

| - What does the label ADHD tell us   - ADHD indicates that there are difficulties in class   - ADHD indicates that you should be more understanding   - ADHD gives standard solution for large group   - ADHD also indicates positive traits   - ADHD can also mean that there are internalizing problems   - ADHD is not fixed and can change with time and context   - ADHD says a lot   - ADHD does not say much   - ADHD has a strong stereotype - Positive effects of diagnosis   - Diagnosis provides guidance   - Diagnosis gives legitimacy to what is going on   - Diagnosis gives relief   - Diagnosis provides an explanation   - Diagnosis helps with explanation and understanding in class   - Diagnosis helps with understanding and accepting oneself   - Diagnosis should ensure acceptance   - Diagnosis takes away guilt   - DSM provides a common language   - Strong stereotypes make diagnoses convenient - Negative effects of diagnosis   - ADHD as a cause, leads to medication as a solution   - ADHD can legitimize rejection   - ADHD is not diagnosed for the individual, but for the class   - DSM suggests that symptoms are fixed   - Instructions for use for ADHD often too short-sighted   - Young people sometimes use diagnosis as an excuse   - Labels can be negative or unpleasant   - Labels can have a stigmatizing effect   - Labels are used quickly and easily   - People can also hide behind a diagnosis - How should we use ADHD   - ADHD as a point of attention   - ADHD as a general manual   - ADHD as a means of communication   - ADHD as an explanation for problems   - ADHD acts as a kind of roadmap   - ADHD indicates that extra effort is needed   - ADHD should help get funding and support   - ADHD should never be seen as the sole cause or explanation   - ADHD should make you more curious   - Thinking in boxes is a quick way to understand someone   - Diagnoses are a convenient approach to a large class   - Health care workers must consciously use or not use the label   - Parents and children often want diagnosis   - Room for fluidity only later in the diagnostic process. - Individual is at the center   - Don't tar ADHD with one brush   - Importance considering whether or not label helps   - Look at what individual help is required.   - Action-oriented is on the rise   - Holistic view of people is valuable   - Looking at individual with a common language   - Looking at the individual instead of a label   - Looking at what does justice to the person   - Label is just one piece of the puzzle   - Label is the starting point for getting to know the person   - Label should not determine how we look at someone   - Listening to children and young people - What is already going well in the system   - In principle, classification is no longer necessary for care   - Care providers carefully consider labels   - Listening and hearing are important skills of care providers   - Changes in dealing with labels over time   - Reduction in demand for and use of labels - What else needs to be done in the system   - Schools   - Families   - Assistance   - Society   - Individuals | - Wat vertelt het label ADHD ons   - ADHD geeft aan dat het moeilijk gaat in de klas   - ADHD geeft aan dat je begrip moet hebben   - ADHD geeft standaardoplossing voor grote groep   - ADHD indiceert ook positieve eigenschappen   - ADHD kan ook zeggen dat er internaliserende problemen zijn   - ADHD staat niet vast en kan veranderen met de tijd en context   - ADHD zegt heel veel   - ADHD zegt niet veel   - Sterk stereotiep bij ADHD - Positieve effecten van diagnose   - Diagnose geeft houvast   - Diagnose geeft legitimering van wat er aan de hand is   - Diagnose geeft opluchting   - Diagnose geeft verklaring   - Diagnose helpt bij uitleg aan en begrip vragen van klas   - Diagnose helpt met zelfkennis en begrip   - Diagnose moet zorgen voor acceptatie   - Diagnose neem schuld weg   - DSM zorgt voor gemeenschappelijke taal   - Sterke stereotypes maakt diagnoses handzaam - Negatieve effecten van diagnose   - ADHD als oorzaak, leidt tot medicatie als oplossing   - ADHD kan afwijzing legitimeren   - ADHD wordt niet gediagnosticeerd voor individu, maar voor klas   - DSM suggereert dat symptomen vaststaan   - Gebruiksaanwijzing ADHD vaak te kortzichtig   - Jongeren gebruiken diagnose soms als excuus   - Labels kunnen negatief of onprettig zijn   - Labels kunnen stigmatiserend werken   - Labels worden snel en makkelijk gebruikt   - Mensen kunnen zelf ook achter diagnose verschuilen - Hoe moeten we ADHD inzetten   - ADHD als aandachtspunt   - ADHD als algemene gebruiksaanwijzing of handvat   - ADHD als communicatiemiddel   - ADHD als verklaring voor problemen   - ADHD fungeert als een soort roadmap   - ADHD geeft aan dat extra inzet nodig is   - ADHD moet helpen bij krijgen financiering en ondersteuning   - ADHD moet nooit worden gezien als de enige oorzaak of verklaring   - ADHD zou nieuwsgieriger moeten maken   - Denken in hokjes is een snelle manier om iemand te begrijpen   - Diagnoses zijn een handige benadering van een grote klas   - Hulpverleners moeten label bewust wel of niet gebruiken   - Ouders en kinderen vaak behoefte aan diagnose   - Ruimte voor fluïditeit pas later in de het diagnostisch proces. - Individu staat centraal   - ADHD niet over een kam te scheren   - Belang overweging of label wel of niet helpt   - Bij individu kijken waarbij hulp nodig is   - Handelingsgericht is sterk in opkomst   - Holistische kijk mensen is waardevol   - Kijken naar individu met een gemeenschappelijke taal   - Kijken naar individu in plaats van label   - Kijken naar wat recht doet aan de persoon   - Label is slechts een stukje van de puzzel   - Label is startpunt voor het leren kennen van de persoon   - Label moet niet te bepalend zijn voor hoe naar iemand kijkt   - Luisteren naar kinderen en jongeren - Wat gaat er al goed in het systeem   - Classificatie in principe niet meer nodig voor basiszorg   - Labels worden door hulpverleners goed overwogen   - Vaardigheid luisteren en horen bij hulpverleners zeer belangrijk   - Verandering in omgang met labels over tijd   - Vermindering in vraag naar en gebruik van labels - Wat moet er anders in het systeem   - Scholen   - Gezinnen   - Hulpverlening   - Maatschappij   - Individu |
| --- | --- |

**Focus group 7: Adolescents**

| - What does ADHD mean   - What is ADHD   - Symptoms of ADHD   - People with ADHD need…   - What does ADHD say about a person   - ADHD runs in the family - Medication   - Positive sides of medication   - Negative sides of medication   - Interest in each other's experiences with medication   - Medication used to be necessary, but not anymore   - Trial and error which medication works - Sharing ADHD with other people   - ADHD diagnosis automatically incorporated in the system   - ADHD diagnosis is shared with friends   - ADHD is not discussed in daily life   - ADHD is often discussed in daily life   - Don't share ADHD until you know someone well   - Need to control who knows or doesn't know you have ADHD   - Sharing experiences of ADHD with friends   - Sometimes nice and sometimes not nice that people know about ADHD - Why were you diagnosed   - Diagnosis due to difficulties at school   - Diagnosis recommended by the teacher   - Visiting a specialist for ADHD testing   - Without a diagnosis, no medications - What's fun about ADHD   - ADHD also has a fun side; funny and spontaneous behavior   - A positive trait of ADHD is making other people laugh - What's is not fun about ADHD   - ADHD behavior sometimes perceived as disturbing by the environment   - ADHD is seen as something abnormal   - ADHD is sometimes seen as something pathetic   - ADHD is sometimes found to be irritating   - Rejecting diagnosis because of negative connotation   - There are prejudices associated with ADHD   - Impulsivity usually a disadvantage - How are we using the label well   - Due to ADHD, undesirable behavior is understood differently   - Diagnosis makes people take you into account more   - No worries about negative connotation ADHD   - Label provides explanation and guidance   - Label should act as a tool   - People have different expectations because of diagnosis   - Advantages of diagnosis outweigh the disadvantages - What is not going well in using the label   - Different treatment by environment because of diagnosis   - Teachers should be better attuned to the needs of students   - Experience of doubts about authenticity diagnosis by environment   - The term ADHD is overused   - The term ADHD is often linked to negative things   - The term ADHD should be used strictly as a diagnosis   - Due to diagnosis, people distance themselves more   - Adults should respond less negatively to ADHD behavior - Every individual is different   - Only experience of hyperactivity in the head   - Experiencing control of the symptoms   - Everyone with an ADHD diagnosis is different   - Differences in how hyperactivity is experienced   - Finding ways to deal with hyperactivity. | - Wat betekent ADHD   - Wat is ADHD   - Symptomen van ADHD   - Mensen met ADHD hebben nodig…   - Wat zegt ADHD over een persoon   - ADHD zit in de familie - Medicatie   - Positieve kanten medicatie   - Negatieve kanten medicatie   - Interesse in elkaars ervaringen met medicatie   - Medicatie vroeger nodig, maar nu niet meer   - Trial en error welke medicatie werkt - Het delen van ADHD met andere mensen   - ADHD-diagnose automatisch in het systeem opgenomen   - ADHD-diagnose wordt gedeeld met vrienden   - ADHD komt niet ter sprake in het dagelijkse leven   - ADHD komt vaak ter sprake in het dagelijkse leven   - ADHD niet delen totdat je iemand goed kent   - Behoefte aan controle over wie wel of niet weet dat je ADHD hebt   - Delen ervaringen ADHD met vrienden   - Soms fijn en soms niet fijn dat mensen weten over ADHD - Waarom heb je de diagnose gekregen   - Diagnose door moeilijkheden op school   - Diagnose op aanraden juf   - Naar specialist voor onderzoek ADHD   - Zonder diagnose, geen medicijnen - Wat is leuk aan ADHD   - ADHD heeft ook een leuke kant; grappig en spontaan gedrag   - Positieve eigenschap van ADHD is andere mensen aan het lachen maken - Wat is niet leuk aan ADHD   - ADHD-gedrag soms als storend ervaring door de omgeving   - ADHD wordt gezien als iets niet normaals   - ADHD wordt soms gezien als iets zieligs   - ADHD wordt soms irritant gevonden   - Diagnose afstoten vanwege negatieve connotatie   - Er zitten vooroordelen aan ADHD vast   - Impulsiviteit meestal nadelig - Wat gaat er goed in het gebruik van het label   - Door ADHD wordt ongewenst gedrag anders begrepen   - Door diagnose wordt er meer rekening gehouden   - Geen zorgen over negatieve connotatie ADHD   - Label geeft uitleg en houvast   - Label zou moeten fungeren als hulpmiddel   - Mensen hebben andere verwachtingen vanwege diagnose   - Voordelen van diagnose zijn groter dan de nadelen - Wat gaat er niet goed in het gebruik van het label   - Andere behandeling door omgeving vanwege diagnose   - Docenten moeten beter afstemmen op behoeftes leerlingen   - Ervaring van twijfels over echtheid diagnose door omgeving   - Term ADHD wordt te veel gebruikt   - Term ADHD wordt vaak gekoppeld aan negatieve dingen   - Term ADHD zou strikt als diagnose moeten worden gebruikt   - Vanwege diagnose wordt er afstandelijker gedaan   - Volwassenen moeten minder negatief reageren op ADHD-gedrag - Ieder individu is anders   - Alleen ervaring van drukte in het hoofd   - Ervaring controle over de symptomen   - Iedereen met een ADHD-diagnose is anders   - Verschillen in hoe drukte wordt ervaren   - Vinden van manier om met drukte om te gaan. |
| --- | --- |
